# Mechanistic and spectroscopic characterization of human CYP17A1 in Nanodiscs

**DOI:** 10.1016/j.jinorgbio.2025.113211

**Published:** 2026-01-07

**Authors:** Ilia G. Denisov, Yilin Liu, Piotr J. Mak, Stephen G. Sligar

**Affiliations:** a Departments of Biochemistry, University of Illinois at Urbana-Champaign, Urbana, IL 61801, United States of America; b Department of Chemistry, University of Akron, Akron, OH 44325, United States of America; c Department of Chemistry, Saint Louis University, Saint Louis, MO 63103, United States of America; d Chemistry, University of Illinois at Urbana-Champaign, Urbana, IL 61801, United States of America

**Keywords:** Cytochrome P450, Peroxide reactivity, Carbon-carbon lyase mechanism, Steroid, metabolism, Raman spectroscopy, Low temperature trapping

## Abstract

The human cytochrome P450 CYP17A1 plays a critical role in the production of steroid hormones, converting pregnenolone to dehydroepiandrosterone and progesterone to androstenedione. Sequential reactions catalyzed by CYP17A1 are hydroxylation at C17 position, followed by C17 – C20 carbon-carbon bond scission. The mechanism of the lyase reaction is still debated, with two proposed reaction pathways favoring either a peroxo-(Compond 0) or iron-oxo (Compound 1) driven catalysis. In this review we summarize the results obtained through collaboration between the Sligar laboratory at University of Illinois and the Kincaid laboratory at Marquette University over the last 15 years. We used a combination of spectroscopic and functional studies of human CYP17A1 incorporated in lipid Nanodiscs, mimicking the native membrane environment, to dissect the elementary steps of P450 reaction cycle and characterize the iron-oxygen intermediates in the presence of substrates for both reactions catalyzed by CYP17A1. In addition, we used the mutations E305G and T306A to probe the effect of perturbing the proton delivery required for the formation of Compound 1, but not for Compound 0, and the mutation N202S involved in substrate positioning at the active site. Resonance Raman spectra, in combination with cryo-radiolytic reduction of the oxy-complex of CYP17A1, provided a detailed picture of hydrogen bonding and protonation of peroxo- and hydroperoxo- intermediates and identified a new transient hemiketal complex on the peroxo-driven pathway of lyase reaction. These results consistently demonstrated the predominant role of the peroxo-driven catalysis for the lyase reaction in CYP17A1 incorporated in lipid Nanodiscs.

## 1. Introduction

**Fig. 1.**
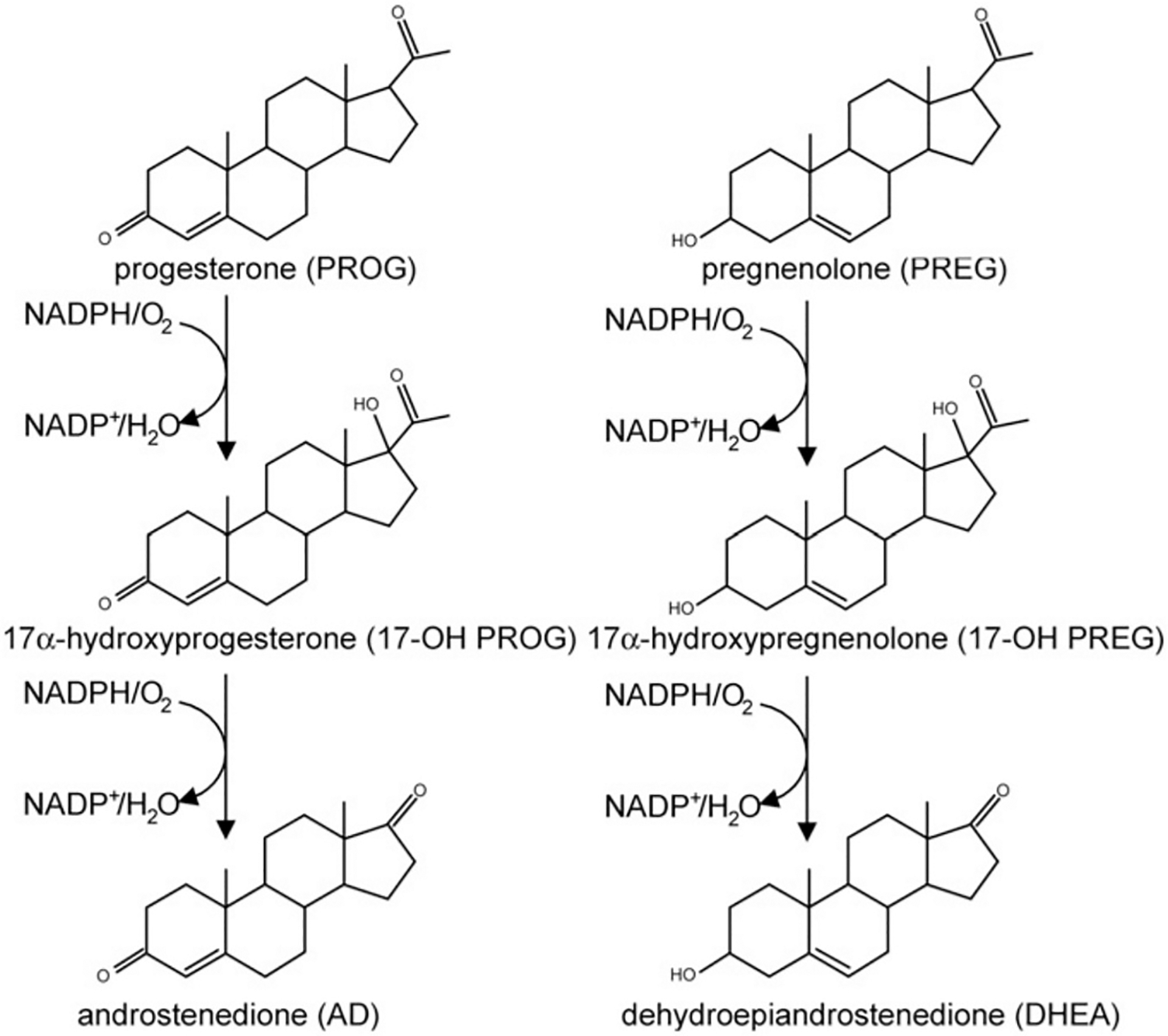
Reactions catalyzed by human CYP17A1 with progesterone (PROG) and pregnenolone (PREG) as substrates.

Cytochromes P450 play an essential role in steroid hormone biosynthesis in all vertebrates [1]. One key player, CYP17A1, is critical in catalysis of two-step conversions from pregnenolone (PREG) and progesterone (PROG) to the dehydroepiandrosterone and androstenedione, Fig. 1. These reactions involve hydroxylation at the C17 position, followed by the C–C scission step between C17 and C20 carbon atoms [2,3]. Importantly, the second reaction is dramatically accelerated in the presence of cytochrome *b*5 (Cytb_5_) [4–6]. While the first reaction is typical for P450 enzymes and proceeds via a Compound I (Cpd 1) driven hydrogen abstraction and oxygen rebound pathway, the mechanism of the C–C lyase reaction has been debated for a long time.

Comparison of hydroxylation and C–C bond scission reaction catalyzed by CYP17A1 reveals significant differences, such as different product formation rates and coupling efficiency (NADPH consumption vs. product formation), substrate preferences (PROG vs. PREG) and also critical role of Cytb_5_ for the lyase reaction [7]. An important difference between hydroxylation and lyase reactions is also observed when kinetic solvent isotope effect (KSIE) is measured. While KSIE is normal for the former (k_H_/k_D_ > 1, as expected for the proton-dependent catalysis), the inverse KSIE with k_H_/k_D_ < 1 is observed for the latter, suggesting that different catalytic mechanisms are involved [8–10]. Mechanistic aspects and involvement of different catalytic species were discussed in favor of peroxo-ferric catalyzed pathway [11,12] and against it [13,14], with one QM/MM study indicating the preference for the former [15]. Other P450 examples for lyase catalysis include CYP19A1 [16–18], CYP51A1 [19,20], CYP199A4 [21–23], CYP2B4 [16], with various conclusions made on the question of Cpd 1 vs. peroxo-ferric intermediate as the main catalytic species. Overall, the preferable pathway for the catalysis of the C–C bond scission in CYP17A1 is apparently determined by the positioning of the substrate relative to the iron-oxygen intermediate, including the presence or absence of the hydrogen bond of C17 hydroxyl group to the proximal oxygen atom of Fe-OO and Fe-OO^−^ intermediate (s), and the ability of P450 enzyme to arrange protonation of the distal oxygen atom for Fe-OOH formation. The substrate orientation turns out to be the most important factor, as indicated by the difference between PREG and PROG. This difference in positioning of substrates was demonstrated by the structural and functional studies of the WT and N202S mutant CYP17A1 [24].

All mammalian cytochromes P450 are bound to the membrane via a single N-terminal transmembrane helix, and the F-G loop together with G’ helix are also involved in the protein-lipid interactions. The redox partner flavoprotein cytochrome P450 reductase (CPR) is similarly connected to the membrane via one transmembrane helix, as well as cytochrome *b*5. These interactions with the membrane impose restrictions on the mutual orientation of P450 enzymes and their redox partners, which stabilize their interactions in the functionally important charge-transfer complexes [25,26]. Interactions of transmembrane helices of cytochrome P450 and redox partners inside the lipid bilayer play an essential role in protein-protein functional interactions, as shown experimentally [27] and by molecular dynamics (MD) simulations [28,29]. Therefore, reconstitution systems used for structural and functional studies of human cytochromes P450 should be based on the utilization of lipid membrane incorporated proteins, such as microsomal preparations, lipid vesicles, bicelles, or lipid Nanodiscs, as these systems preserve the role of lipid bilayer in the protein-protein interactions. For Nanodiscs, the precise control of lipid composition and monomeric state of cytochrome P450 incorporated into the membrane provide a well characterized stable structurally homogeneous preparation, which can be used in cryospectroscopic experiments [30,31] and stopped-flow studies for detailed kinetic measurements [32–38], as well as in functional studies of the steady-state kinetics of product formation [9,10,38–45].

X-ray structures of CYP17A1 with bound substrates and inhibitors provided a detailed picture of functionally important interactions defining orientation of substrates with respect to the catalytically active heme-oxygen intermediates [46–48]. Significantly, the role of N202 side chain was revealed in adjusting the position of PREG and PROG substrates via hydrogen bonding to the C3-OH group of the former and the keto-group at the same position for the latter. In addition, positions of 17-hydroxypregnenolone were slightly different in different molecules in the crystal, with some of them closer to the heme iron and well suited to form a hydrogen bond between C17 hydroxyl group and proximal oxygen of the Fe-OO^−^ peroxo intermediates, which optimizes formation of the hemiketal intermediate on the C–C bond scission catalytic pathway [7,44].

However, all structures of CYP17A1 to date are obtained for the ferric state of the protein, and no X-ray structure of oxy-complex is available. As it was shown for the CYP101A1 [49,50], reduction of cytochrome P450 and oxygen binding can be accompanied by subtle local conformational changes and appearance of water molecules, which potentially can be important for the efficient proton transfer required for catalysis. Therefore, direct structural information about positioning of the substrate with respect to the iron-oxygen intermediates, and about hydrogen bonding and proton transfer should be obtained from alternative experimental methods, mostly various spectroscopic studies of P450 catalytic intermediates stabilized at low temperatures in frozen solutions at 77 K.

### 1.1. Kinetic solvent isotope effect as a mechanistic probe

Important insight into the nature of the alternative catalytic pathway of the lyase reaction was provided by measurement of kinetic solvent isotope effects (KSIE). Mechanistic studies based on comparison of the rates of enzyme catalysis in H_2_O and in D_2_O buffers were introduced by Showen [51,52] and Cleland [53,54] and described for several enzymes where proton transfer was rate-limiting and essential for the catalytic step. Normally, these reactions are faster in H_2_O than in D_2_O because of slightly higher energy of the D–O bond in deuterated water and therefore slower proton transfer events. However, for some reactions the inverse KSIE was observed, when product forming rates were faster in D_2_O [55,56]. The most straightforward interpretation of such results is the presence of two pathways, with one of them requiring protonation from solvent water, and another one independent of protonation. If the product is formed on the pathway which does not require protonation, and the proton-dependent pathway is unproductive, the observed rate of product formation in such system will be higher in D_2_O than in H_2_O. Such examples were observed for tyrosine hydroxylase [55].

**Fig. 2.**
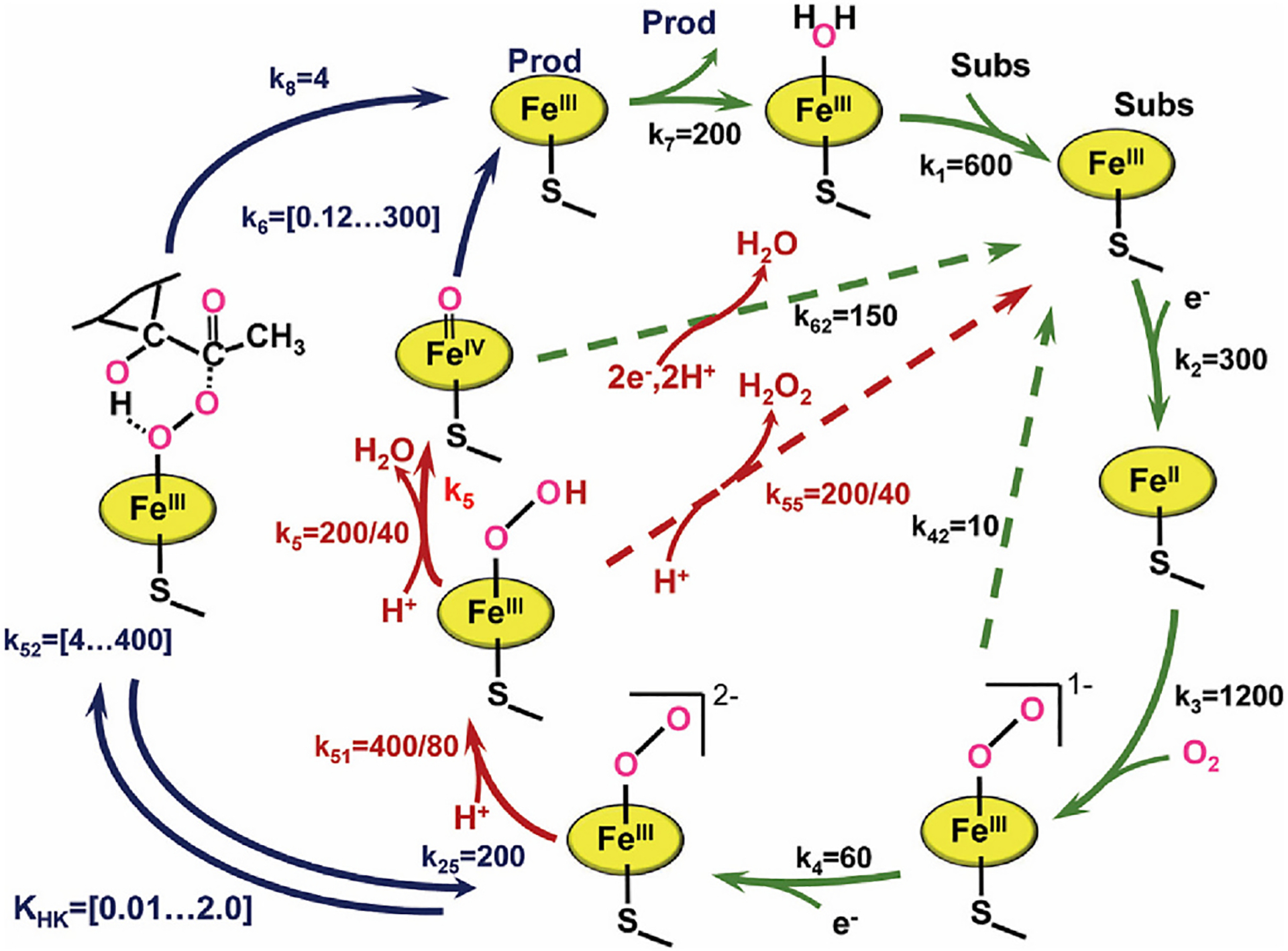
Catalytic cycle of CYP17A1 with two alternative product forming pathways. Hydroxylation catalyzed by Cpd 1 requires two protons from solvent water, while C–C bond scission catalyzed by peroxo-ferric intermediate is proton independent. Shown are the rates of elementary reaction steps used in the modeling of steady-state kinetics of product formation [8].

**Fig. 3.**
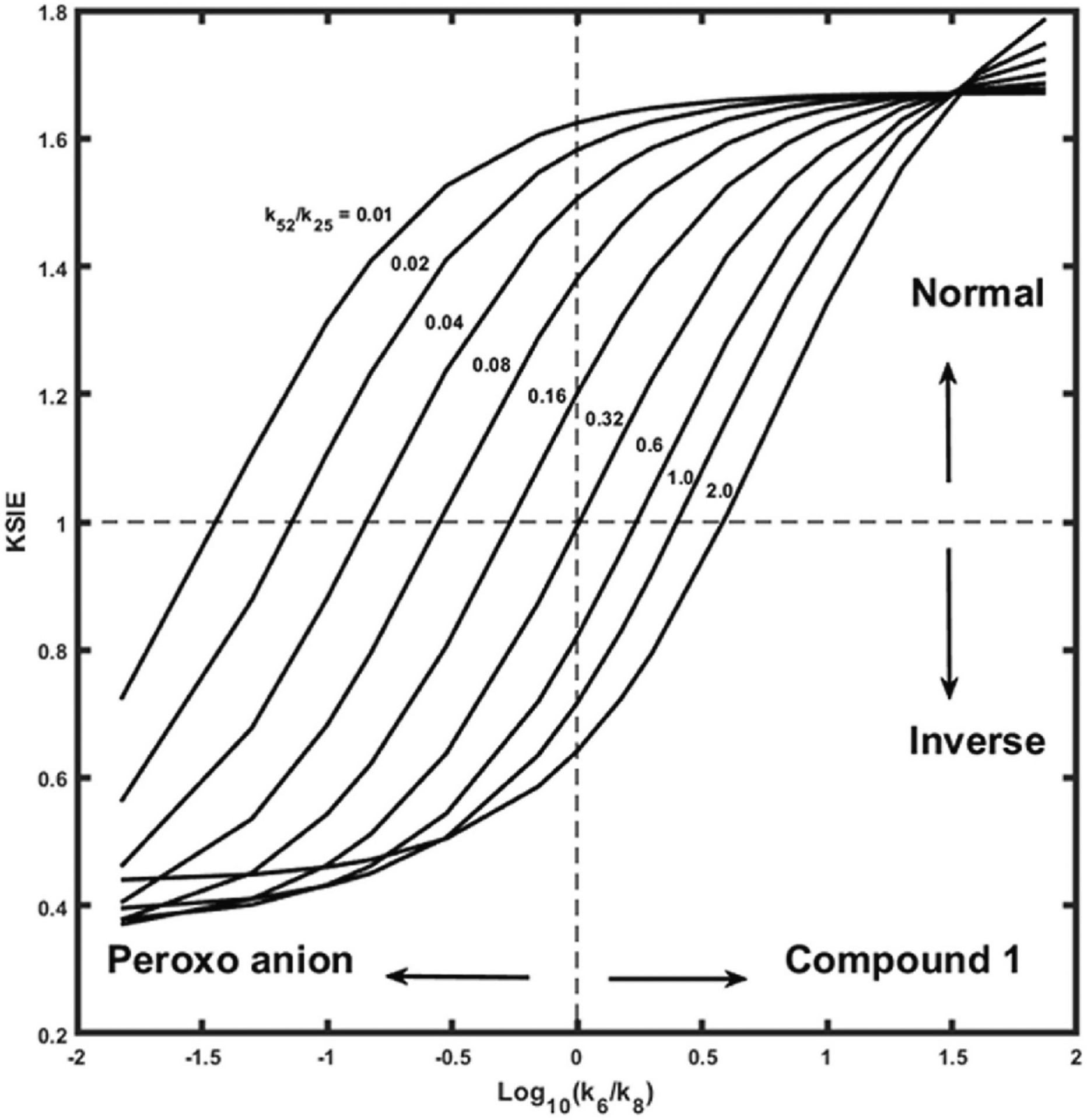
Results of kinetic modeling of KSIE measured for CYP17A1 catalysis with product forming rates via Cpd 1 k_6_ and via peroxo-ferric intermediates k_8_ with different values of the equilibrium constant for hemiketal formation (see Fig. 2 for definition). For predominantly Cpd 1 product forming pathway, when k_6_ is 10–40 times higher than k_8_, the normal KSIE >1.3 is observed, while for inverse KSIE <0.8 the hemiketal pathway is dominant with k_8_ significantly larger than k_6_ [8].

Analysis of KSIE for the identification of the catalytic mechanism of CYP17A1 catalyzed C–C scission in 17-hydroxyprogesterone (17-OH PROG) and 17-hydroxypregnenolone (17-OH PREG) is complemented by the comparison to available KSIE measurements for hydroxylation reaction, catalyzed by the same CYP17A1 enzyme under identical conditions. For hydroxylation reaction catalyzed by Cpd 1 by plethora of cytochromes P450, the normal KSIE in the range 1.3–2.0 is observed, which is consistent with two protonation steps of peroxo-ferric and hydropero-ferric intermediates, obligatory steps to generate Cpd 1. The same normal KSIE was measured for hydroxylation of PREG and PROG by CYP17A1 and several mutants [9,24,41,42,45,57]. However, for the lyase reaction, an inverse KSIE was always observed with both 17-OH PREG and 17-OH PROG, suggesting the alternative, protonation independent catalytic pathway (Fig. 2). Moreover, inverse KSIE not only suggests the product formation via an alternative unprotonated catalytic intermediate, it also indicates that Cpd 1 pathway is not productive with lyase 17-hydroxylated substrates, and that it serves as the uncoupling channel. For this unproductive channel via Cpd 1 the steady-state reaction flux is significantly higher in H_2_O because of faster protonation than in D_2_O, and therefore the productive channel contributes smaller fraction into the total NADPH consumption. In D_2_O the situation is different, with smaller flux via Cpd 1 proton-dependent unproductive channel, and more product is formed via proton-independent peroxo-ferric catalyzed path. This partitioning was analyzed in detail in our recent publication [8], where we show that experimentally observed inverse KSIE is not compatible with the predominant Cpd 1 catalyzed product formation (Fig. 3).

### 1.2. Effect of E305G and T306A on lyase activity of CYP17A1

Another strong indication of different catalytic mechanisms of hydroxylation and lyase reactions in CYP17A1 emerges from comparison of effects of functionally important mutations at the acid-alcohol pair, which is critically important for efficient proton delivery and Cpd 1 formation in P450 enzymatic function [49,50,58–64]. Both E305G and T306A mutations significantly inhibit product formation rates of PREG and PROG hydroxylation by CYP17A1, while C–C bond scission rates remain relatively unaffected [10,41,42]. The strong inhibiting effect of mutations in these positions is common for many P450 enzymes, where Cpd 1 is the main catalytic species responsible for hydroxylation reactions. The fact that the lyase reaction catalyzed by CYP17A1 is not significantly perturbed by E305G and T306A mutations adds more evidence in favor of an alternative catalytic mechanism, which is not dependent upon the proton delivery to the iron-oxygen intermediates.

An additional experimental observation demonstrating mechanistic difference between hydroxylation and lyase reactions catalyzed by CYP17A1 is the much stronger pH dependence of the latter. Both reactions are faster at pH 7.0 than at pH 7.8, but for lyase reaction the pH dependence is more pronounced in the E305G mutant to such extent, that C–C scission is becoming significantly faster than in the wild type CYP17A1 at pH 7.0. This is unusual, since in general the opposite is true, and hydroxylation reaction is considerably faster because of higher uncoupling at the lyase step.

### 1.3. Role of cytochrome b5 in CYP17A1 catalysis

A very important aspect of the CYP17A1 catalyzed lyase reaction is the critical role of its interaction with cytochrome *b*5. It was shown previously that in the absence of Cytb_5_ formation of products is strongly inhibited or not detected at all [4,65–68]. The role of cytochrome *b*5 as a redox partner is attributed to the second electron transfer and acceleration of reduction of oxygenated CYP17A1, thus preventing uncoupling of NADPH redox equivalents through autoxidation [69,70]. The same mechanistic role of Cytb_5_ was demonstrated previously for CYP2B4 using stopped flow and single turnover experiments [71,72] and cryospectroscopy [73], as reviewed [74]. Another possible contribution of Cytb_5_ is via allosteric effect on the iron-sulfur bond in CYP17A1, which may favor catalysis [75], or by affecting the substrate positioning with respect to the catalytic intermediate, although the details of such interactions are not clear at this time.

As it was first shown for CYP2B4, the oxy-complex of cytochrome P450 was reduced to peroxo-ferric or hydroperoxo-ferric intermediate significantly faster in the presence of reduced cytochrome *b*5. Combining these results with the single-turnover experiments provided the overall phenomenological conclusion of the significant role of cytochrome *b*5 in improving coupling ratio of the general NADPH consumption to the product forming rate [65,70]. It was also shown that CPR and Cytb_5_ compete for the same binding site on P450 molecule, although more detailed mechanistic interpretation of the role of Cytb_5_ was not attempted in these studies of CYP2B4 – cytochrome *b*5 system.

Later it was shown that involvement of Cytb_5_ in efficient transfer of second electron to the oxygenated CYP17A1 was similar to those CYP2B4 studies, as studied experimentally in Nanodiscs using stopped flow spectroscopy [69]. Reduced CYP17A1 saturated with 17-OH PREG was co-incorporated with reduced CPR, or reduced cytochrome *b*5, and mixed with oxygen saturated buffer, in order to monitor fast oxygen binding to the P450 enzyme, followed by disappearance of oxy-complex. The rate of autoxidation of CYP17A1 without redox partners was 0.02 s^−1^ at 25° C, while the rate of electron transfer from Cytb_5_ to the oxygenated CYP17A1 was ~0.9 s^−1^, significantly faster than from CPR (~0.1 s^−1^). When both CPR and Cytb_5_ were co-incorporated in Nanodiscs with CYP17A1 in equimolar ratios, the observed rate of disappearance of oxy-complex was 1.3 s^−1^, indicating the preferential interaction of cytochrome *b*5 and faster second electron transfer in ternary complex. The functional competence of cytochrome *b*5 as the donor of the second electron for CYP17A1 was also confirmed by comparison of product forming rates with the redox inactive cytochrome *b*5 reconstituted with Mn-protoporphyrin IX [38]. The rate of DHEA production from 17-OH PREG as a substrate was 5-fold accelerated with native cytochrome *b*5, while no acceleration was observed with Mn-substituted b5. These observations explain improved coupling and activation of CYP17A1 in the presence of b5, and suggest the same role played by cytochrome *b*5 with other P450 enzymes.

Our results can be compared with the recently reported kinetic study of the first and second electron transfer rates from CPR and cytochrome *b*5 to CYP17A1 saturated with substrates [70]. These experiments were performed in mixtures of ~100 μM DLPC with the addition of the detergent Anapoe-X-305; no significant difference between rates of second electron transfer from b5 and from CPR was found. The main conclusion, i.e. the ability of reduced Cytb_5_ to deliver second electron and form product in lyase reaction, is the same in both studies.

Resonance Raman (rR) spectroscopy provides an unprecedented insight into the hydrogen bonding of the dioxygen moiety in CYP17A1 with substrates bound at the active site, and the positioning of the substrate predisposed for catalysis. Using rR spectroscopy combined with cryoradiolytic reduction of oxygenated CYP17A1 the detailed information on the catalytically active intermediates was obtained, as described below in this review.

### 1.4. Resonance Raman spectroscopy of CYP17A1 catalytic intermediates

Stabilization of P450 intermediates at cryogenic temperatures is critically important for spectroscopic studies, because of fast autoxidation of oxygenated heme enzymes and fleeting nature of peroxo-ferric and hydroperoxo-ferric intermediates. The methods of cryoradiolytic reduction and experimental details of spectroscopic characterization of intermediates in P450 enzymes have been described [31,43,44,76–80]. The samples of CYP17A1 in Nanodiscs saturated with substrate of choice were oxygenated with pure ^16^O_2_ or ^18^O_2_ in H_2_O and D_2_O buffers and quickly frozen in liquid nitrogen to stabilize oxy-complexes. rR spectra were measured in spinning NMR tubes immersed in liquid nitrogen. For cryoradiolytic reduction these samples were irradiated by gamma rays from Cobalt-60 source while immersed in liquid nitrogen, as described [76,81]. Irradiation generates free electrons from frozen water-glycerol solvent, and these electrons reduce heme-oxygen complex to form peroxo-ferric and hydroperoxo-ferric intermediates, which are stable at 77 K. These intermediates were characterized by rR spectroscopy at 77 K, and after annealing at 165–190 K, to allow gradual protonation and formation of hydroperoxo-ferric intermediate. Further annealing resulted in formation of catalytically active Cpd1 intermediate, or hemiketal intermediate, depending on the substrate suited for hydroxylation or for lyase reaction. Functional competence of these intermediates obtained by cryoradiolysis was confirmed by direct measurements of product formation in these single turnover experiments by CYP17A1 without redox partner [82,83], similar to other cryoreduced P450 enzymes, such as CYP101 and mutants [49,84–86], CYP101D1 [87], CYP11A1 [88], CYP2B4 [89] and NOS [90].

### 1.5. Resonance Raman studies of substrate binding effects on the active site of ferric CYP17A1

**Fig. 4.**
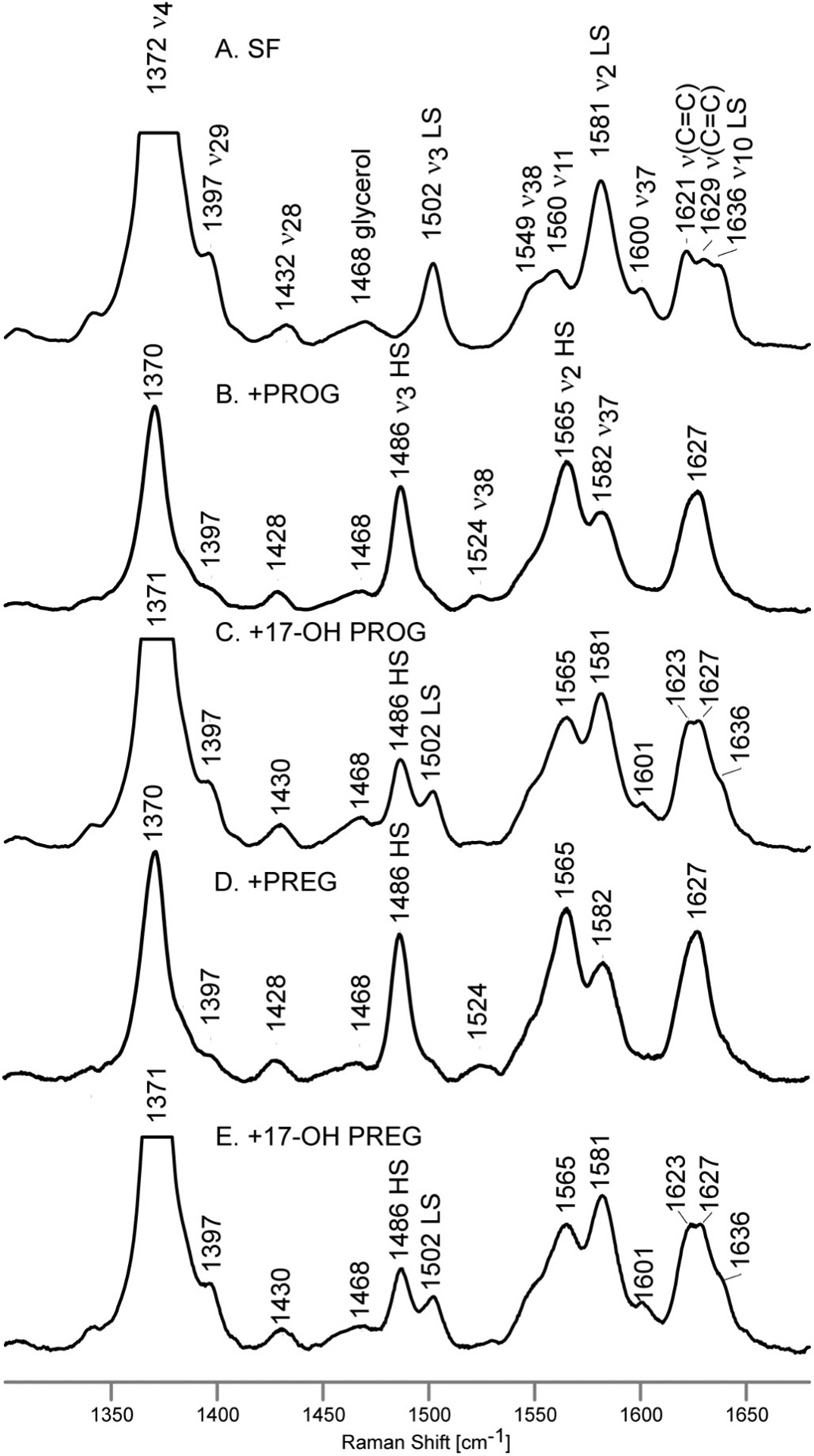
The high frequency rR data of ferric ND:CYP17A1 in substrate-free state (A) and with the following substrates: PROG (B), 17-OH PROG (C), PREG (D) and 17-OH PREG (E). The spectra were normalized to the glycerol mode observed at 1468 cm^−1^ [93].

The active site structure of CYP17A1 in ferric state was interrogated using rR spectroscopy to provide insight into differential substrate-induced alterations caused by the presence of four of the substrates shown in Fig. 1. The rR spectrum of substrate-free CYP17A1 (Fig. 4) exhibits the ν_4_ oxidation state marker and the so-called spin state marker bands, ν_3_, ν_2_ and ν_10_, indicative of a pure low spin 6-coordinated state (LS6c), reflecting a water molecule coordination. [91–93]

The addition of PROG and PREG substrates results in significant changes in the rR spectrum. The spin state markers are consistent with a pure high spin 5-coordinated (HS5c) state [80,91,92,94–99]. On the other hand, the binding of hydroxylated substrates, 17-OH PROG and 17-OH PREG, leads to only a partial spin state conversion, as seen by the presence of bands characteristic for both LS6c and HS5c states. Such partial spin-state conversions upon binding of certain substrates have been observed previously for other cytochrome P450 enzymes and are most often attributed to incomplete expulsion of the distal pocket water cluster and coordination of a residual water molecule [91,92].

**Fig. 5.**
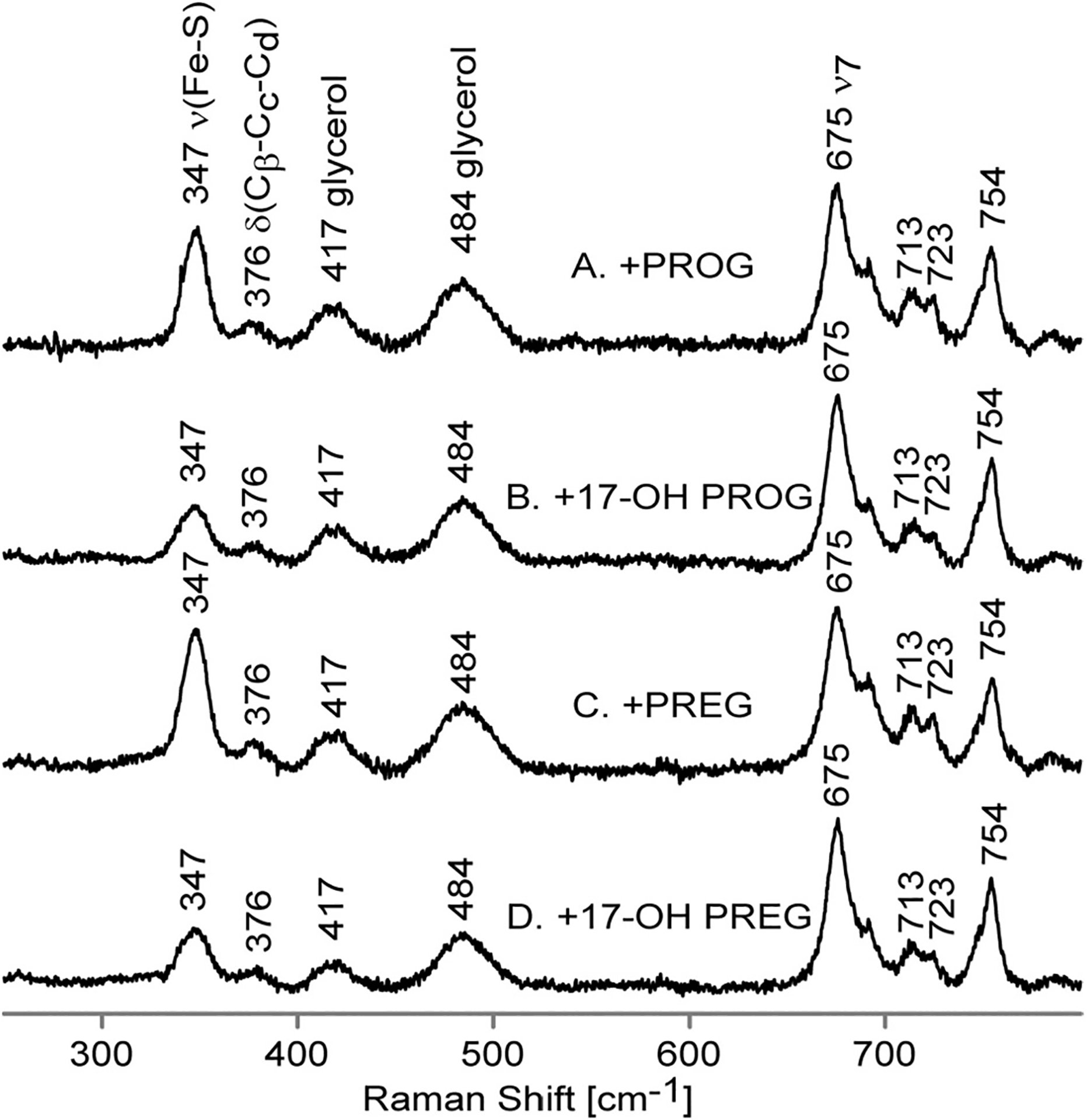
The low frequency rR data of ferric CYP17A1 with the following substrates: PROG (A), 17-OH PROG (B), PREG (C) and 17-OH PREG (D). The spectra were normalized to the *ν*_7_ mode observed at 675 cm^−1^. Excitation laser line was 356.7 nm [93].

The low-frequency rR spectra provide valuable information on the disposition of the heme peripheral groups and out-of-plane distortions of the heme macrocycle [100–102] as well as the modes associated with binding of endogenous and exogenous heme axial ligands [91,103–106]. Interestingly, the addition of any of the four substrates caused no significant changes in the modes associated with the propionate and vinyl bending modes, indicating that hydrogen bonding to the propionate groups is unchanged upon binding of any of these substrates, nor is the alteration in geometry of vinyl groups. However, substrate binding did result in an activation of the out-of-plane (OOP) modes. Furthermore, it was determined that linkage between the heme iron and its endogenous thiolate ligand remains identical in the presence of any of the four substrates, i.e., the substrate structure does not affect the strength of the Fe–S trans-axial linkage, as judged by the observation of the *ν*(Fe–S) stretching mode at 347 cm^−1^ in each case (Fig. 5). Consequently, it is reasonable to propose that changes in the modes associated with exogenous axial ligands, such as the OO or CO fragments, are exclusively attributable to distal side interactions.

In summary, careful analysis of the data acquired for the ferric forms shows that binding of substrate causes variable degrees of conversion from the LS to HS state, with the non-hydroxylated PROG and PREG yielding almost complete HS form, while the two hydroxylated substrates (17-OH PROG and 17-OH PREG) give incomplete conversion to HS.

### 1.6. Differential hydrogen bonding in CYP17A1 oxy complexes

The oxy adducts are the last relatively stable intermediates in the enzymatic cycle of cytochromes P450. All three ν(Fe–O), ν(O–O), and δ(FeOO) modes associated with the inherently bent Fe-O-O fragment are effectively enhanced in rR spectra of these thiolate ligated proteins. The ν(Fe–O) and ν(O–O) stretching modes are typically detected in 517–541 cm^−1^ and 1125–1140 cm^−1^ regions [91]. The relatively low frequency of the ν(O–O) mode as well as Mössbauer quadrupole splitting for the iron, indicate that the correct formulation of the oxy adduct is a ferric superoxide species, instead of a ferrous dioxygen form [107]. The changes in the vibrational frequencies of these modes reflect alterations in the Fe–O and O–O bond strengths or the Fe-O-O angle. The geometry of the Fe-O-O fragment can be affected by H-bonding or electric fields of amino acid side chains on the distal side of the heme pocket, as well as steric contacts that can distort the electronic structure of the heme macrocycle. It is noted that rR spectroscopy is particularly well-suited for studying the oxy adducts of heme proteins, especially since their comprehensive characterization is often unattainable by other spectroscopic methods; e.g., the oxy adducts are diamagnetic and EPR silent and the *ν*(Fe–O) stretching mode is not observed in the IR spectra while the ν(O–O) mode appears in an area convoluted with many overlapping modes that complicate its assignment.

In addition, it has been shown by experimental rR studies of heme proteins and model hemes and computational density functional theory (DFT), that the distal active site electrostatic interactions with the oxygen atoms of the Fe-O^P^-O^T^ fragment (where O^P^ indicates the proximal, or iron-adjacent atom and O^T^ stands for terminal, or outer oxygen atom) can also be relatively easily deciphered, as they shift the *ν*(Fe–O) and *ν*(O–O) modes in well-established ways [79,108–112]. The H-bonding to O^P^ results in a positive ν(Fe–O)/ν(O–O) correlation, while H-bonding to O^T^ generally results in a negative/inverse relationship of these modes. In other words, the H-bonding to the inner O^P^ atom downshifts the ν(Fe–O) and ν(O–O) frequencies simultaneously by increasing the anti-bonding character of the Fe–O and O–O bonds to give greater sp^2^ character to O^P^. Conversely, donation of H-bonds to the outer O^T^ atom increases backbonding, weakening the O–O bond and strengthening the Fe–O bond, resulting in shifts of the ν(O–O) and ν(Fe–O) modes to lower and higher frequencies, respectively.

**Fig. 6.**
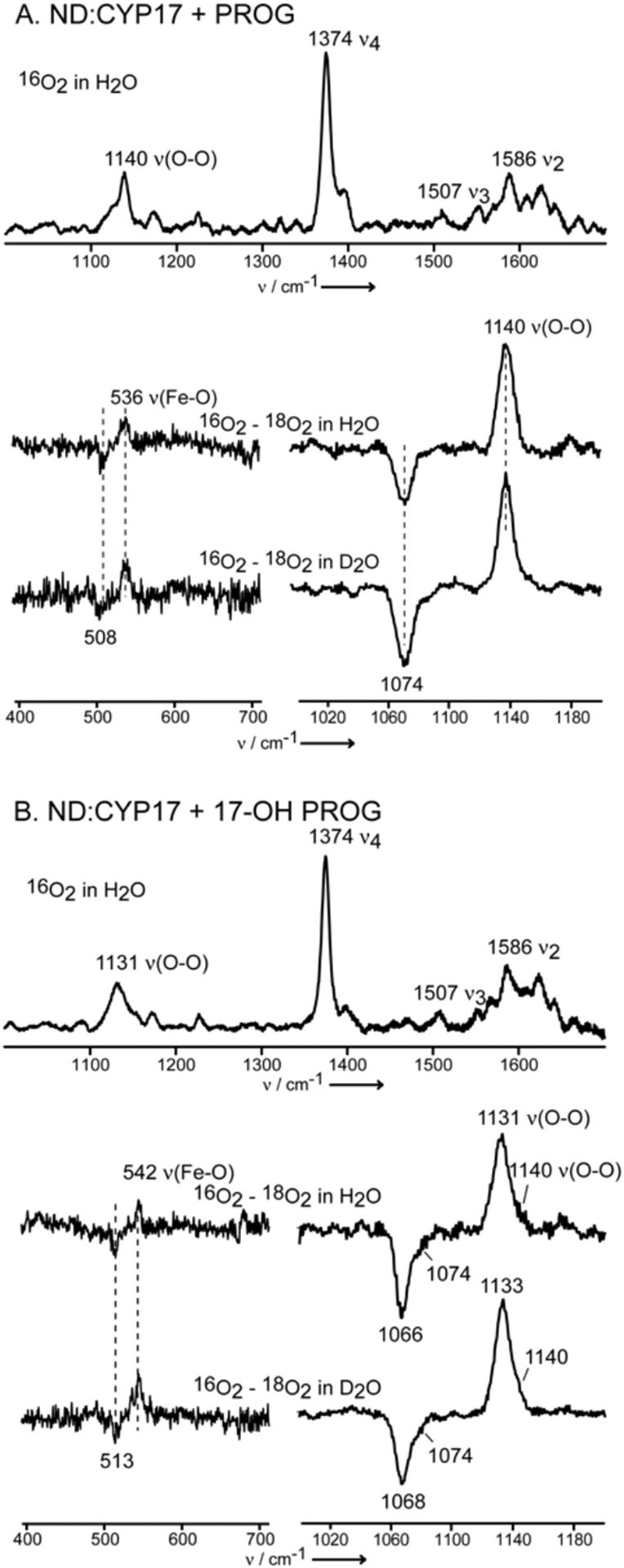
The rR spectra of PROG and 17-OH PROG bound ^16^O_2_ adducts of CYP17A1 in H_2_O buffer (panel A and B, respectively). The lower section of each panel shows ^16^O_2_–^18^O_2_ difference plots in H_2_O (upper) and D_2_O (lower) buffers [43].

**Fig. 7.**
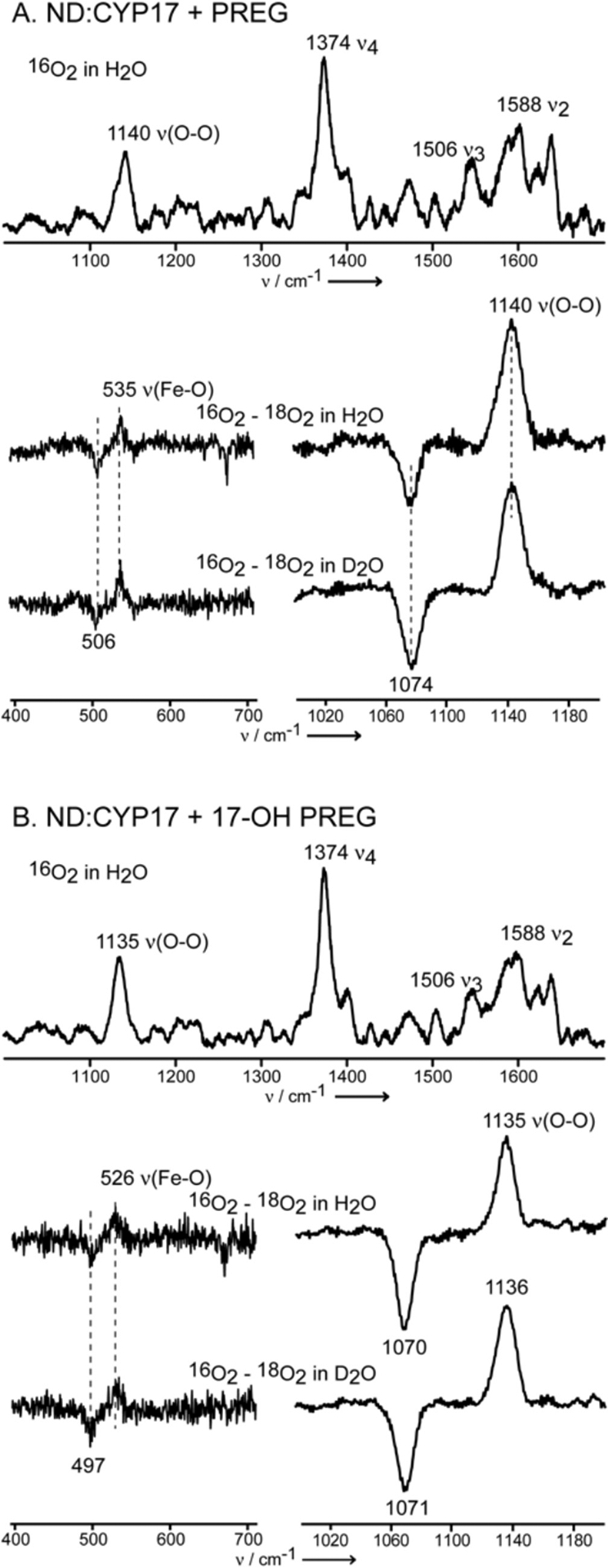
The rR spectra of PREG and 17-OH PREG bound ^16^O_2_ adducts of CYP17A1 in H_2_O buffer (panel A and B respectively). The lower section of each panel shows ^16^O_2_–^18^O_2_ difference plots in H_2_O (upper) and D_2_O (lower) buffers [43].

Figs. 6 and 7 (top), show the absolute rR spectra obtained for the ^16^O_2_ adduct of CYP17A1 in the presence of PROG, 17-OH PROG, PREG and 17-OH PREG substrates. The structure-sensitive heme modes, such as *ν*_4_, ν_3_, and ν_2_ indicate that the protein in all four samples is in 6cLS state, as typically observed for oxy adducts of cytochromes P450.

In the spectrum of PROG-bound sample (Fig. 6, A) the *ν*(^16^O–^16^O) and the corresponding ν(Fe–O) stretching mode appear at 1140 cm^−1^ and 536 cm^−1^ as confirmed by the uncluttered ^16^O_2_/^18^O_2_ difference traces, directly below. These frequencies are typical for the Fe-O-O fragment with weak H-bonding interactions to P450 active site residues [79,109].^18,19^ The rR spectrum obtained for the ^16^O_2_ adduct of CYP17A1 bound to 17-OH PROG (Fig. 6, B) reveals that the *ν*(^16^O–^16^O) mode appears ~9 cm^−1^ lower at 1131 cm^−1^. The lowering of the *ν*(O–O) mode is correlated with a corresponding 6 cm^−1^ frequency increase of the ν(Fe–O) band relative to its value in the PROG-bound enzyme. It is also noted that the ν(^16^O–^16^O) mode shifts 2 cm^−1^ to a higher frequency in D_2_O solutions [111], indicating H-bonding interaction to the newly introduced C_17_-OH(D) functionality.

Corresponding rR spectra and difference traces for the PREG and 17-OH PREG-bound enzymes are shown in Fig. 7. Similarly to PROG-bound CYP17, the *ν*(O–O) and ν(Fe–O) modes of the PREG-bound enzyme are observed near 1140 and 535 cm^−1^ with no evidence for H-bonding. The 17-OH PREG bound spectrum (Fig. 7, B) reveals that the ν(O–O) mode is shifted down by only 5 cm^−1^ compared with its value for the PREG-bound form. Interestingly, the ν(Fe–O) stretching mode is observed at 526 cm^−1^, exhibiting a 9 cm^−1^ shift to lower frequency compared to the value observed for the sample containing non-H-bonding PREG. It needs to be emphasized that the introduction of the 17-OH group causes downshifts of the ν(O–O) modes for both 17-OH PROG and 17-OH PREG, however, the corresponding ν(Fe–O) modes shift in opposite directions; i.e., the 17-OH PROG yields a 6 cm^−1^ upshift while the 17-OH PREG shows a 9 cm^−1^ downshift. Since it was shown previously that the trans Fe–S bond strength is identical for all four substrates, the effects on the Fe-O-O fragment arise mainly from distal side interactions.

**Fig. 8.**
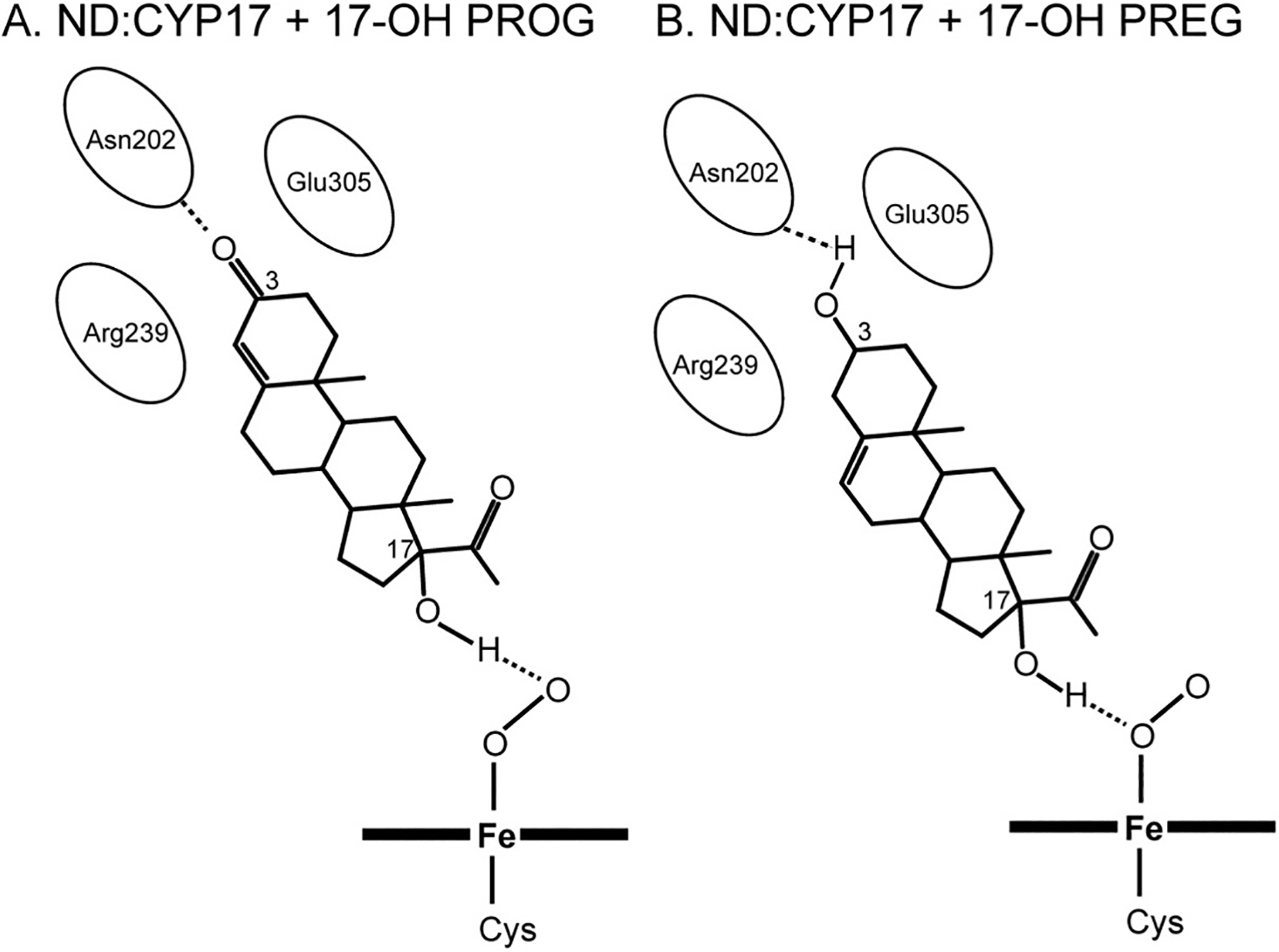
Graphical representation of human CYP17A1 protein substrate interaction derived from these rR data [43].

These rR data provide decisive evidence that the two hydroxylated substrates interact at opposite ends of the Fe-O-O fragment, as depicted in Fig. 8, which can lead to crucial implications for CYP17A1 function; i.e., it is reasonable to assume that these interactions will dictate the preference for the hydroxylation and lyase pathways. It was argued before that H-bonding to O^T^ are expected to promote O–O bond cleavage and Compound I formation, whereas the H—O^P^ interaction should prolong the lifetime of the ferric peroxo-intermediate that is proposed to be the active intermediate in the lyase reaction [113,114]. Indeed, such differences in O^T^ vs O^P^ H-bonding interactions have been invoked for the nitric oxide synthase reaction; in the first cycle, the arginine substrate is suggested to provide a H—O^T^ interaction and facilitate Compound I formation, whereas in the second cycle the hydroxylated substrate (N^G^-hydroxy-l-arginine) is believed to provide a H—O^P^ interaction and proceed through a peroxo-intermediate [111,112,115]. In summary, this single difference at a relatively remote site of the substrate is sufficient to alter the H-bonding interactions with the critical Fe-O-O fragment in such a way as to dictate its predisposition towards one of two alternative reaction pathways.

### 1.7. Detection and characterization of cryoradiolytically reduced unstable intermediates of WT CYP17A1 with PREG and 17-OH PREG bound

The formation and decay of the reactive intermediates encountered in the C–C bond cleavage stage of androgen biosynthesis were monitored by optical spectroscopy. The oxy adducts were prepared in a solution of buffer containing 60% glycerol along with saturating concentrations of the appropriate substrate at −30 °C. Anaerobic reduction of the protein, followed by the addition of oxygen gas to the sample at low temperature, resulted in the formation of oxy-ferrous protein with nearly 100% yield. The samples were cooled to 77 K and then exposed to a 4 Mrad dose from a ^60^Co source to generate hydrated electrons, which can migrate at 77 K to produce the initial peroxo intermediate. The generation of the following intermediate states was achieved by raising the temperature from liquid nitrogen temperatures to ~200 K while optical absorption spectra were recorded.

**Fig. 9.**
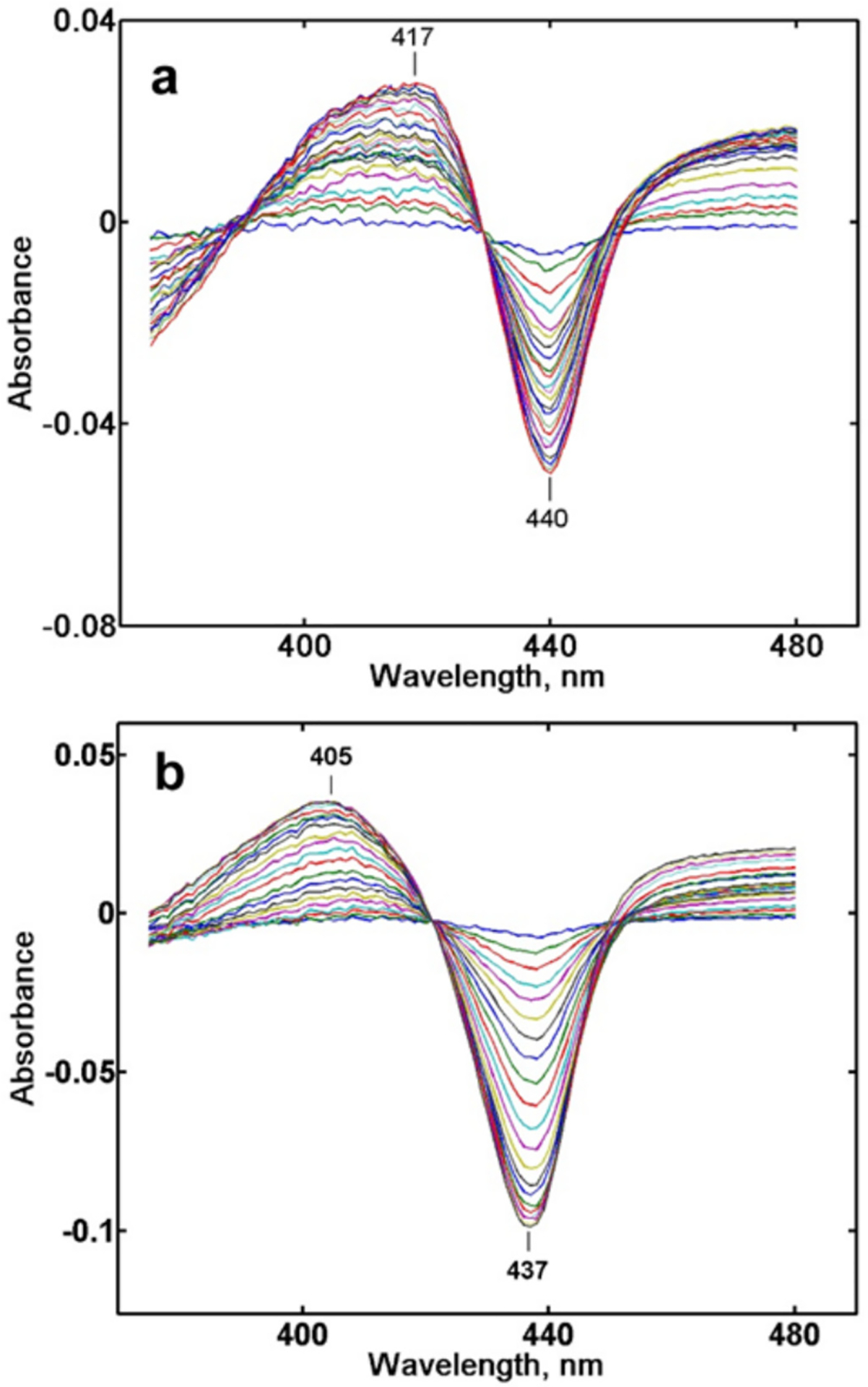
Thermal annealing of peroxo-ferric intermediates monitored by optical absorption spectroscopy in CYP17A1 with different substrates PREG (a) and 17-OH PREG (b). Shown are difference spectra obtained by subtracting of the spectrum at 160 K from the spectra measured at temperatures gradually increasing from 161 K (baseline) to 185 K (maximal difference) [44].

For samples containing PREG or 17-OH PREG (Fig. 9, panels a and b) the presence of a strong (negative) absorption band near 440 nm was detected in the difference spectra, indicating that the initially generated intermediate is the ferric peroxo species (Fig. 9) [79]. Upon annealing to higher temperatures, the behavior of the PREG or 17-OH PREG bound samples starts to differ. The sample containing PREG shows a steady loss of the peroxo intermediate, converting directly to a species that exhibits an absorption spectrum that matches that of the LS ferric state (λ = 417 nm) acquired at 77 K. This behavior is consistent with the rapid progression through the typical O–O bond cleavage cycle, which results in the formation of Compound I and its rapid decay during product formation [77,85]. Remarkably, however, during an identical temperature excursion for the 17-OH PREG-bound sample, the decay of the peroxo-like Soret band at 437 nm was accompanied by the unexpected formation of a new species with a Soret maximum near 405 nm.

**Fig. 10.**
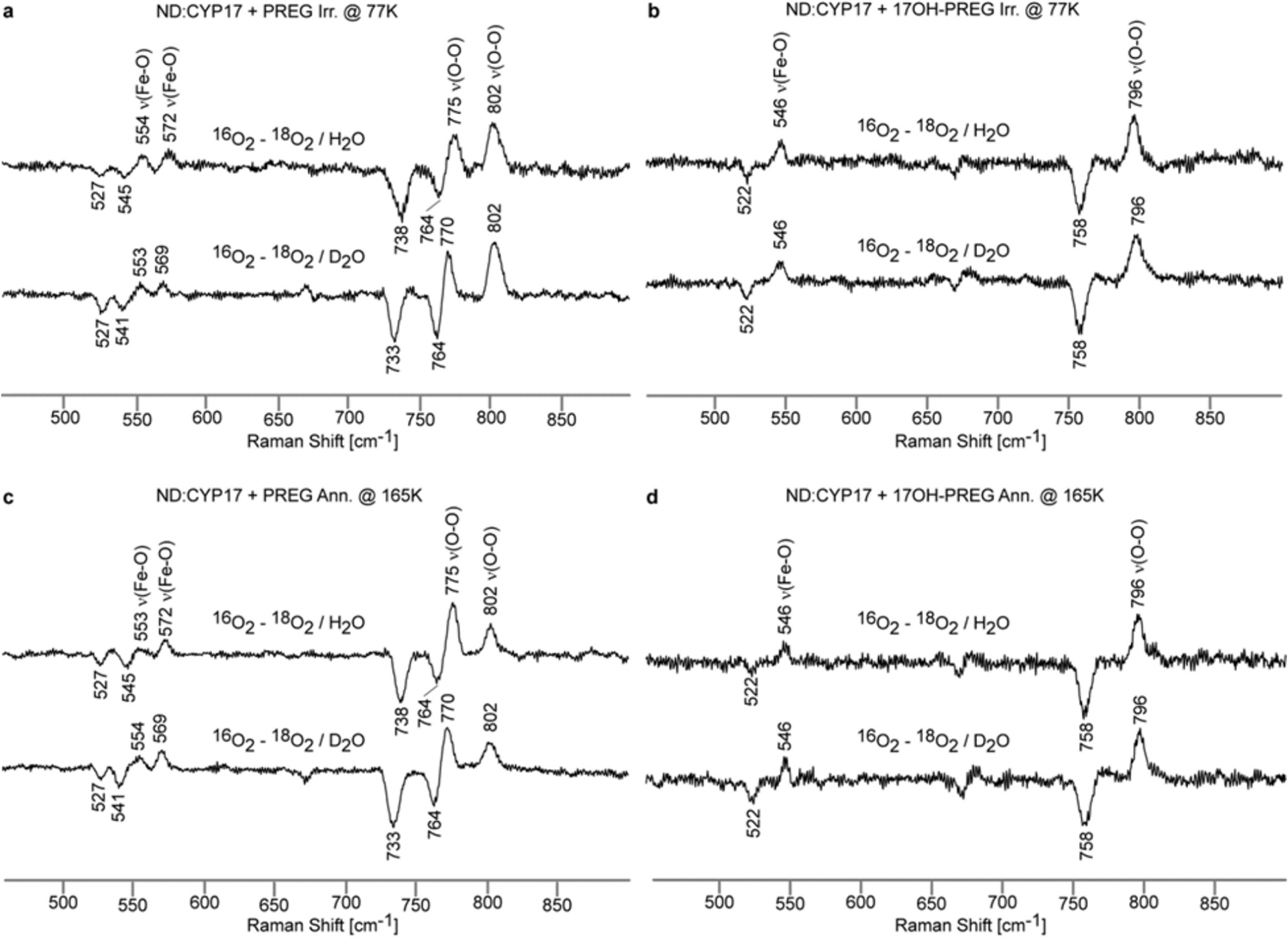
Resonance Raman spectral data for irradiated dioxygen adducts of CYP17A1. The rR ^16^O_2_ - ^18^O_2_ difference traces in H_2_O and D_2_O buffers of irradiated oxy CYP17A1 samples (before annealing) with PREG (a) and 17-OH PREG (b) and corresponding samples after annealing to 165 K (c) and (d). Employing the *isolated* bands for the ^16^O-peroxo (802 cm^−1^) and ^18^O-hydroperoxo (736 cm^−1^) species, the I_736_/I_802_ increases from 0.72 to 1.42. Similar values were obtained using the data from samples prepared with D_2_O buffers (from 0.77 to 1.59) [44].

The ^16^O_2_–^18^O_2_ difference traces for samples containing PREG- and 17-OH PREG substrates in H_2_O and D_2_O buffers at 77 K and 165 K are plotted in Fig. 10. The difference traces for samples containing PREG show two sets of oxygen-isotope sensitive (^16^O_2_/^18^O_2_) modes in the initial cryoreduced samples, indicating the presence of two intermediates. The modes observed at 802 cm^−1^ and 554 cm^−1^ do not exhibit H/D sensitivity and are assigned to the *ν*(^16^O–^16^O2) and ν(Fe–^16^O) stretching frequencies of the ferric peroxo intermediate. The second set of modes at 775 cm^−1^ and 572 cm^−1^ (ν(^16^O–^16^O) and ν(Fe–^16^O) stretching modes, respectively) exhibits a 3–5 cm^−1^ isotopic shift in samples prepared in D_2_O buffers, consistent with their assignment to the hydroperoxo-derivative. The decrease in the intensity of the mode associated with the peroxo intermediate at 165 K, with a concomitant increase in the intensity of the hydroperoxo modes, reflects temperature-assisted protonation of the peroxo species, indicating active site architecture with exceptionally efficient proton transfer in the PREG-bound sample, facilitating formation of Compound I and the classical hydroxylation reaction required to produce 17-OH PREG.

The 17-OH PREG substrate contains an additional H-bonding molecular fragment in the immediate heme environment. The initial product of cryoradiolytic reduction exhibits only one species, with *ν*(^16^O–^16^O) mode at 796 cm^−1^ and the corresponding ν(Fe–^16^O) mode occurring at 546 cm^−1^, which are insensitive to the H/D exchange, confirming the identity of this species as a ferric peroxo-intermediate; however, with a slightly different disposition than that seen for the PREG-bound sample. The much lower frequency of the ν(Fe–^16^O) mode, as compared to the peroxo species of PREG-bound sample, suggests that an H-bonding interaction occurs between the hydroxyl group of the substrate and the proximal oxygen atom of the Fe-O^p^-O^t^ peroxo-fragment; consistent with the earlier work on the oxy adducts [43]. This is an important finding, because such a specifically directed H-bonding interaction to the O^p^ of the peroxo-fragment is expected to facilitate its involvement in the lyase phase of catalysis [113,114]; i.e., this result suggests the 17-OH PREG-bound peroxo-intermediate is poised for attack upon the susceptible electrophilic C_20_ carbon of the bound substrate. Annealing of the 17-OH PREG-bound sample to 165 K shows no evidence for the appearance of any new ^16^O/^18^O sensitive bands (Fig. 10, d).

**Fig. 11.**
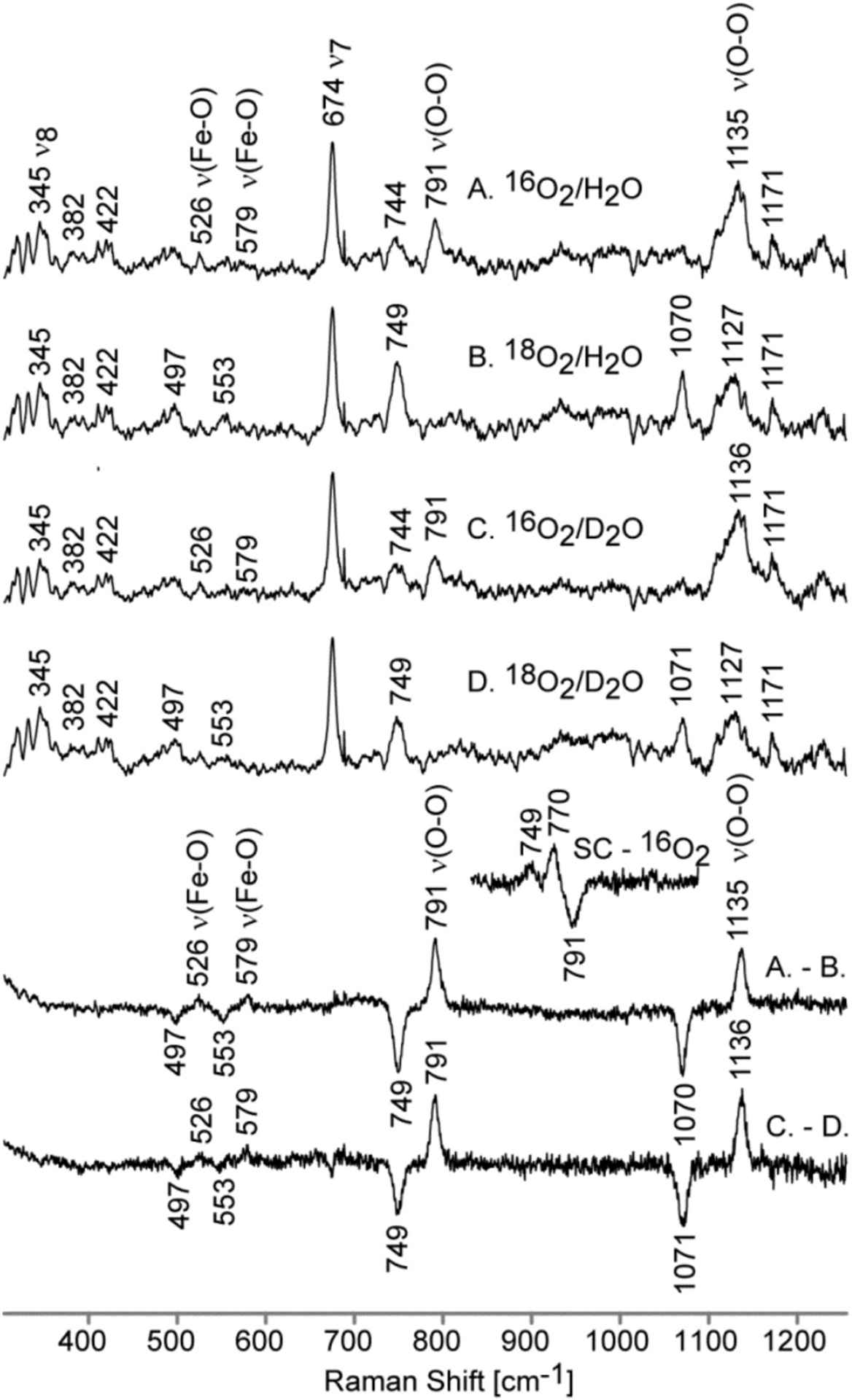
The rR spectral data for irradiated dioxygen adducts of CYP17A1 samples with 17-OH PREG annealed at 190 K. A) ^16^O_2_/H_2_O, B) ^18^O_2_/H_2_O, C) ^16^O_2_/D_2_O, D) ^18^O_2_/D_2_O and their ^16^O_2_ - ^18^O_2_ difference traces; inset shows difference trace of scrambled oxygen (SC) and ^16^O_2_ spectrum. Spectra were measured with a 406 nm excitation line at 77 K; the total collection time for each spectrum was 8–9 h [44].

To characterize the progression of the species arising during the annealing process, the rR spectra were measured with a 406 nm excitation line (Fig. 11), in close resonance with the newly arising second intermediate, which has a Soret maximum near 405 nm, as shown in the optical studies. The rR spectra yielded a new set of strongly enhanced H/D insensitive bands appearing at 791 cm^−1^ and 579 cm^−1^, assigned to *ν*(^16^O–^16^O) and ν(Fe–^16^O), respectively. While the observation of a feature appearing at 791 cm^−1^ is consistent with an intermediate with “peroxo-like” character, it must be noted that this vibrational frequency and ^16^O/^18^O isotopic shifts are close to those expected for the *ν*(Fe = O) mode of a ferryl heme species [116–118]. However, the assignment of this feature to a ferryl species was ruled out by experiments conducted with scrambled oxygen, a (1:2:1) mixture of ^16^O_2_:^16^O^18^O:^18^O_2_, which showed a distinctive three-component difference pattern characteristic of the intermediate containing an intact O–O bond (i.e., ^16^O–^18^O).

The UV–Vis and rR spectral characteristics of the newly detected species closely resemble those of acylperoxo adducts of heme- and non-heme proteins, which possess an Fe-O-O peroxo fragment linked to an oxidized carbon. A blue-shifted Soret maximum at 405 nm (Fig. 9) is consistent with those seen for acylperoxo species derived from meta-chloroperoxybenzoic acid (mCPBA) [119,120] as well as other substituted peroxybenzoic acids [121]. Similarly, Que. and coworkers [122] have also isolated and spectroscopically characterized an acylperoxo derivative of a non-heme iron protein, reporting a value of 792 cm^−1^ for the frequency of the ν(O–O) mode, which is virtually identical to that we observe. Since the ferric peroxo intermediate attacks the C20 carbon of the carbonyl group, we assign this species to peroxo hemiketal intermediate.

In summary, these spectroscopic data obtained through the combined application of cryoreduction, rR and optical spectroscopies, as well as careful isotopic labeling strategies, provide a convincing experimental evidence that the highly directional H-bonding interaction between the hydroxyl group of 17-OH PREG and the proximal oxygen of the ferric peroxoanion leads to the formation of the peroxo hemiketal intermediate, which is poised for an attack on the C_20_ carbon atom of the substrate and facilitating a crucial C–C bond lyase reaction.

### 1.8. PROG and 17-OH PROG bound CYP17

It has been shown that CYP17A1 exhibits a compromised lyase activity in the presence of 17-OH PROG substrate. To reveal the structural basis for this reduced reactivity optical annealing and rR studies were performed on CYP17A1 samples in the presence of PROG and 17-OH PROG substrates. The PROG-bound sample showed results similar to those obtained in a previous study with PREG [44], with a strong negative absorbance associated with the peroxo–/hydroperoxo- species, while increasing temperature led to a new feature near 413 nm [45]. These results are consistent with the formation of Compound I species responsible for the hydroxylation of C17 atom of PROG. Annealing studies of the cryoreduced sample containing 17-OH PROG showed that, around 190 K, the peroxo/hydroperoxo intermediate(s) is almost completely lost, with concomitant appearance of a much weaker absorption near 407 nm. Now following the solid red arrows in Fig. 2, some fraction of the trapped peroxo intermediate can attack the susceptible C_20_ carbonyl to generate a new species shown in the center, whose characteristic absorption band appears near 407 nm.

**Fig. 12.**
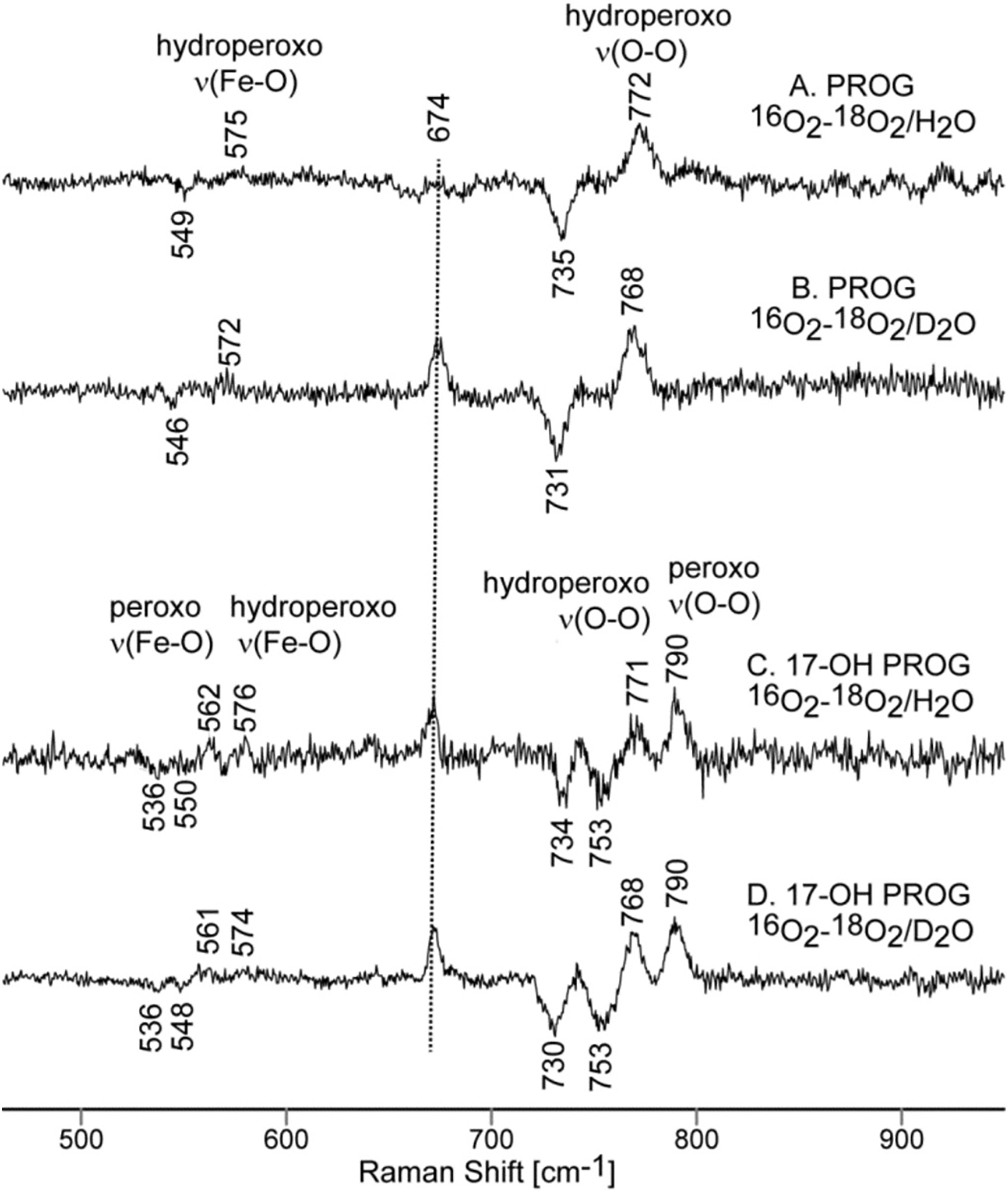
The ^16^O_2_–^18^O_2_ difference traces of irradiated oxygenated CYP17A1 containing PROG (A, B) and 17-OH PROG (C, D) in H_2_O buffer (A, C) and D_2_O buffer (B, D). Spectra were measured at 77 K using 442 nm excitation line and a 4.0–4.5 h total collection time for each rR spectrum. The subtraction was performed in a way to obtain the cleanest difference trace. In some traces, there appears a positive band at around 674 cm^−1^, arising most probably form higher intensity of the ν_7_ mode in the ^16^O_2_ samples, due to a higher amount of residual ferric form in these samples as compared to the ^18^O_2_ samples [45].

The ^16^O_2_–^18^O_2_ difference traces of irradiated CYP17A1 samples with PROG and 17-OH PROG are shown in Fig. 12. The PROG-bound samples show two positive bands at 575 cm^−1^ and 772 cm^−1^, which exhibit a 3–4 cm^−1^ downshift in D_2_O buffer, behavior that prompts their assignment to the *ν*(Fe–O) and ν(O–O) modes of a hydroperoxo intermediate. Interestingly, no evidence was found for the presence of a peroxo intermediate in the PROG-bound sample. The absence of the peroxo intermediate can be explained by invoking a more facile proton-delivery network in the presence of PROG than in the PREG-bound enzyme case, which could also partially account for the lower lyase efficiency of CYP17A1 with this substrate. The cryoreduced 17-OH PROG sample (Fig. 12, traces C and D) exhibits two pairs of *ν*(Fe–O) and ν(O–O) modes. One set is observed at 562 cm^−1^/790 cm^−1^ and does not exhibit H/D, while the second, seen at 576 cm^−1^/771 cm^−1^, shifts in D_2_O buffer. These species are reasonably assigned to a peroxo and a hydroperoxo intermediates, respectively. These results are fairly different than those observed previously for the irradiated 17-OH PREG-bound sample [44], where only one band (showing no H/D sensitivity) was observed at 796 cm^−1^.

The initially formed peroxo-intermediate in the 17-OH PROG sample can either undergo nucleophilic attack on the carbonyl group, the propensity for this process being controlled by the inherent reactivity of this intermediate, or be transformed into the hydroperoxo-intermediate, an event that depends on the efficiency of proton transfer. Equally important, the relative orientation of nearby H-bond donors that interact with the reactive Fe-O-O fragment is a key structural feature that effectively manipulates the reactivity of the peroxo intermediate [43–45]. In fact, the spectral pattern of the peroxo 17-OH PROG sample shows that the ν(Fe–O) mode is at ~8 cm^−1^ higher frequency, a shift consistent with H-bonding of the 17-OH fragment to the *distal* oxygen of the Fe-O-O fragment [45]. Contrary to this, the 17-OH PREG substrate formed H-bonding interactions with the proximal oxygen atom of the Fe-O-O fragment in both the dioxygen and peroxo intermediates [43,44]. Notably, previous computational studies [15,113,114,123] provided strong evidence that H-bonding to the proximal oxygen enhances nucleophilic reactivity, whereas H-bonding to the terminal oxygen diminishes it. Consequently, this data provides evidence for an increased proton transfer efficiency and diminished nucleophilicity of the Fe-O-O fragment of the 17-OH PROG bound sample relative to the sample bound with 17-OH PREG, both factors favoring increased lyase reactivity of CYP17A1 for 17-OH PREG compared to 17-OH PROG, consistent with carefully documented studies of product formation [1,9,10,38].

**Fig. 13.**
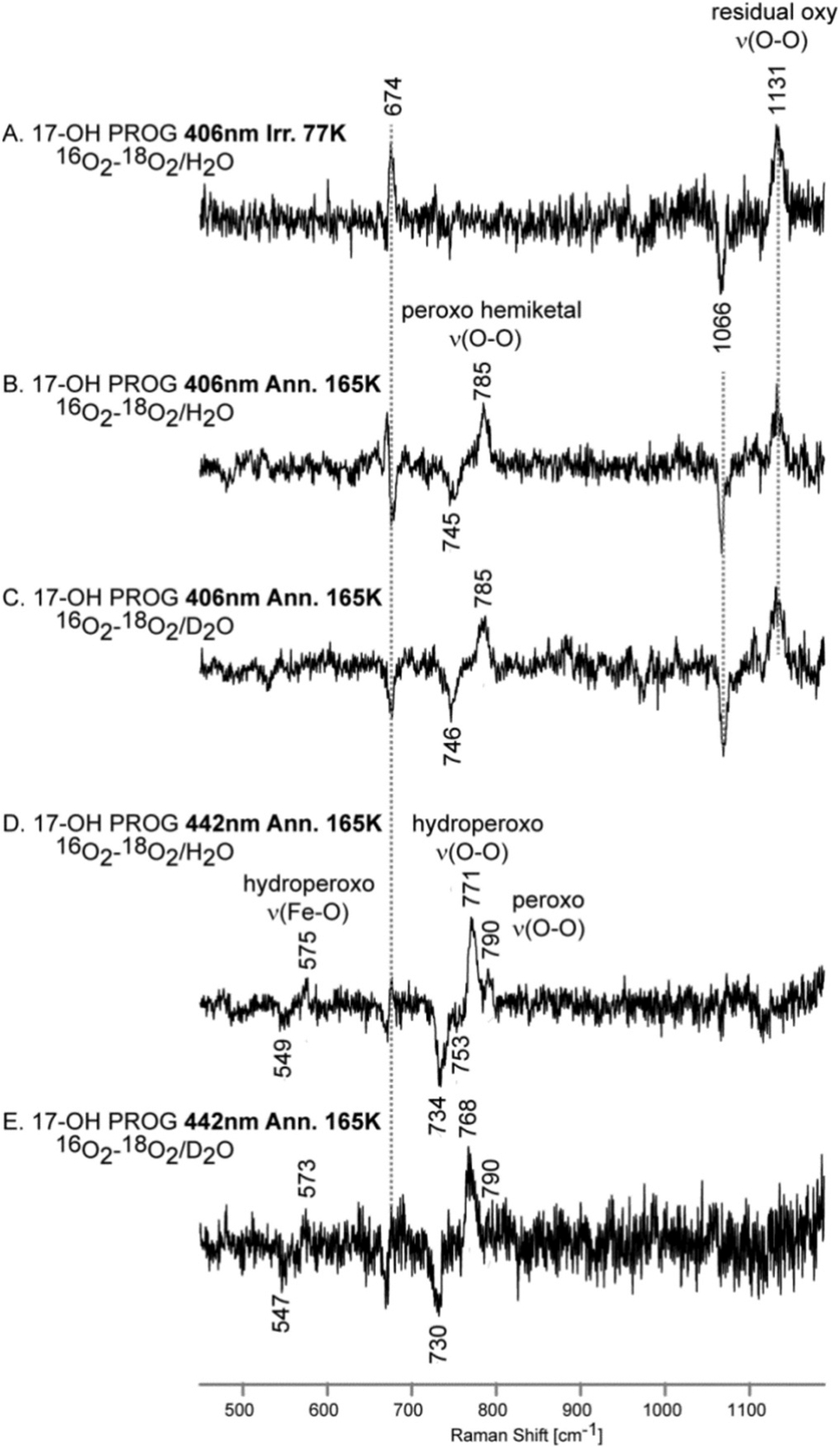
The ^16^O_2_–^18^O_2_ difference trace of irradiated oxygenated CYP17A1 containing 17-OH PROG in H_2_O immediately after irradiation measured with 406 nm excitation line (A), the same H_2_O sample (B) and corresponding sample in D_2_O buffer (C) after annealing at 165 K measured with 406 nm excitation line, the same 165 K annealed samples measured with 442 nm excitation line, in H_2_O (D) and D_2_O (E) buffers. Spectra were measured at 77 K. The difference patterns seen around 106–1140 cm^−1^ in the traces measured with 406 nm excitation line arise from the *ν*(O–O) modes of the residual parent oxy adducts that are strongly enhanced with the 406 nm line [45].

The ^16^O_2_–^18^O_2_ difference spectra of irradiated and annealed 17-OH PROG samples measured with 406 nm excitation are shown in Fig. 13. The spectra recorded at 165 K show formation of H/D insensitive species at 785 cm^−1^ assigned to peroxo hemiketal intermediate [44,45]. The same set of samples (annealed to 165 K), but measured with 442 nm excitation line (traces D and E for H_2_O and D_2_O buffers, respectively), shows that the peroxo species (790 cm^−1^) has nearly disappeared, as compared to the 77 K samples (Fig. 13, traces C and D), while the hydroperoxo species now dominates the rR spectrum, implying that the peroxo-intermediate may react by either forming the hemiketal intermediate or undergoing protonation to form more of the hydroperoxo-intermediate.

**Scheme 1.**
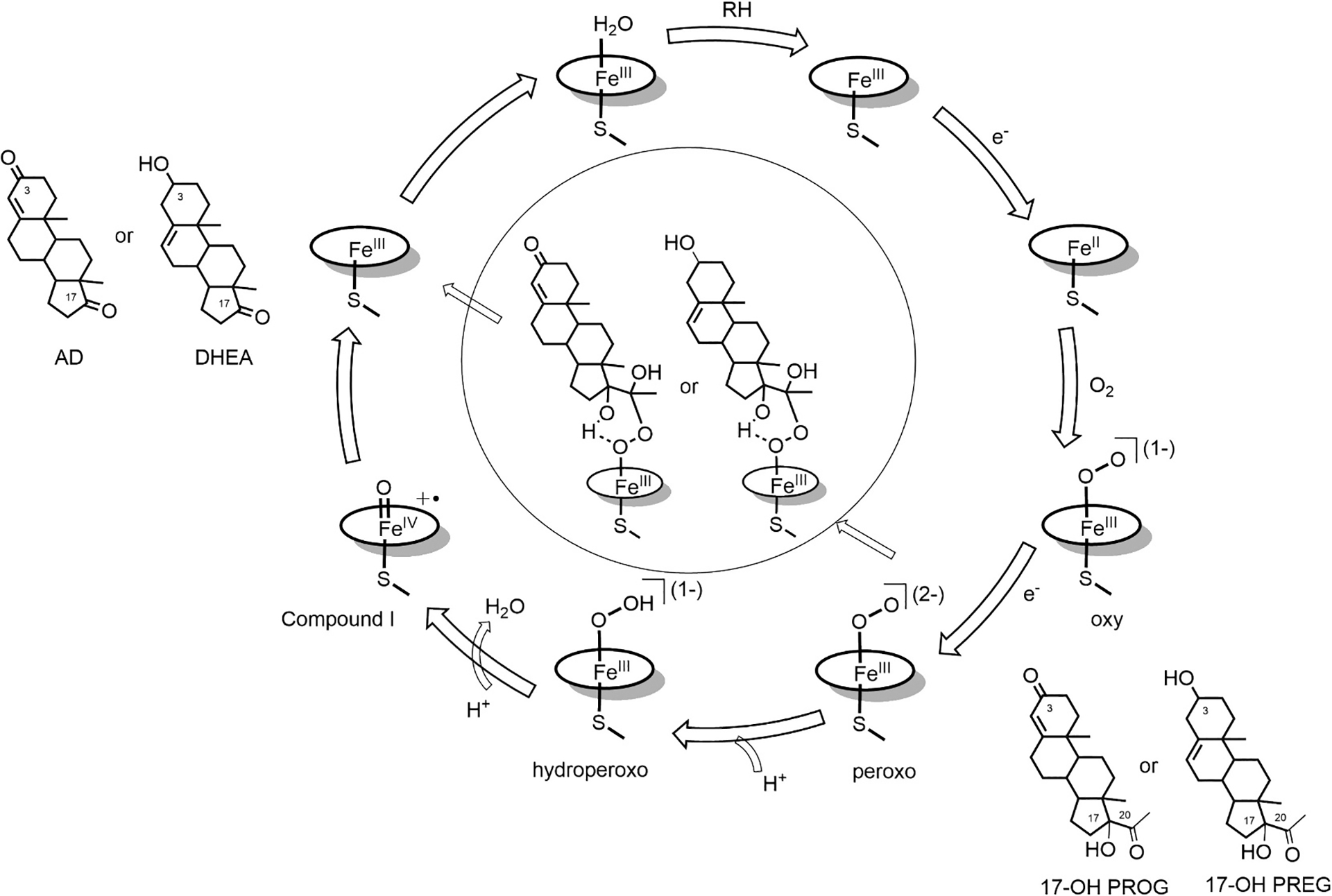
Cytochrome P450 enzymatic cycle and the pathway of the formation of peroxo hemiketal by peroxo attack on C20 of the lyase substrate (17-OH PREG and 17-OH PREG), as shown in the center circle, while the outer circle illustrates the normal P450 catalytic cycle mediated by Compound 1.

Thus, the rR and optical absorption spectroscopy studies provided convincing data consistent with Compound I mediated PROG hydroxylase activity. In case of 17-OH PROG samples, the spectroscopic data convincingly argue for the involvement of a peroxo hemiketal intermediate involved in the lyase reaction sequence. These studies also provide strong evidence for a different active site hydrogen bonding configuration that reflects a more efficient proton delivery network for 17-OH PROG-bound CYP17A1, compared to the complex with 17-OH PREG, revealing an H-bonding interaction with the terminal oxygen of the peroxo fragment, rather than with the proximal oxygen, as is present for 17-OH PREG (Scheme 1). It is proposed that these factors favor diminished lyase activity in the sample containing 17-OH PROG, providing a convincing structural explanation for the significant differences in lyase activity for these substrates.

### 1.9. Key residues in manipulating active site structure and substrate preference of human CYP17A1. The role of N202 residue in substrate positioning and C–C bond scission

The crystallographic studies of PREG and PROG by Scott and coworkers showed that the C-3 substituent of the steroid D-ring is positioned within hydrogen bonding distance to Asn 202 on the F-helix and “might underlie differential substrate metabolism for the 17α hydroxylated substrates” [48]. In a recent study [124] the effects of N202S mutation on both structure and function of CYP17A1 showed that changes in H-bonding between the pregnene-C3 position and residue 202 have a significant impact on product forming rates. The DHEA formation by the N202S mutant is impaired relative to wild-type: 0.12 min^−1^ vs. 0.41 min^−1^, respectively. On the other hand, the product forming rate of AD more than doubled in the mutant protein relative to wild type, increasing from 0.2 min^−1^ to 0.49 min^−1^.

**Fig. 14.**
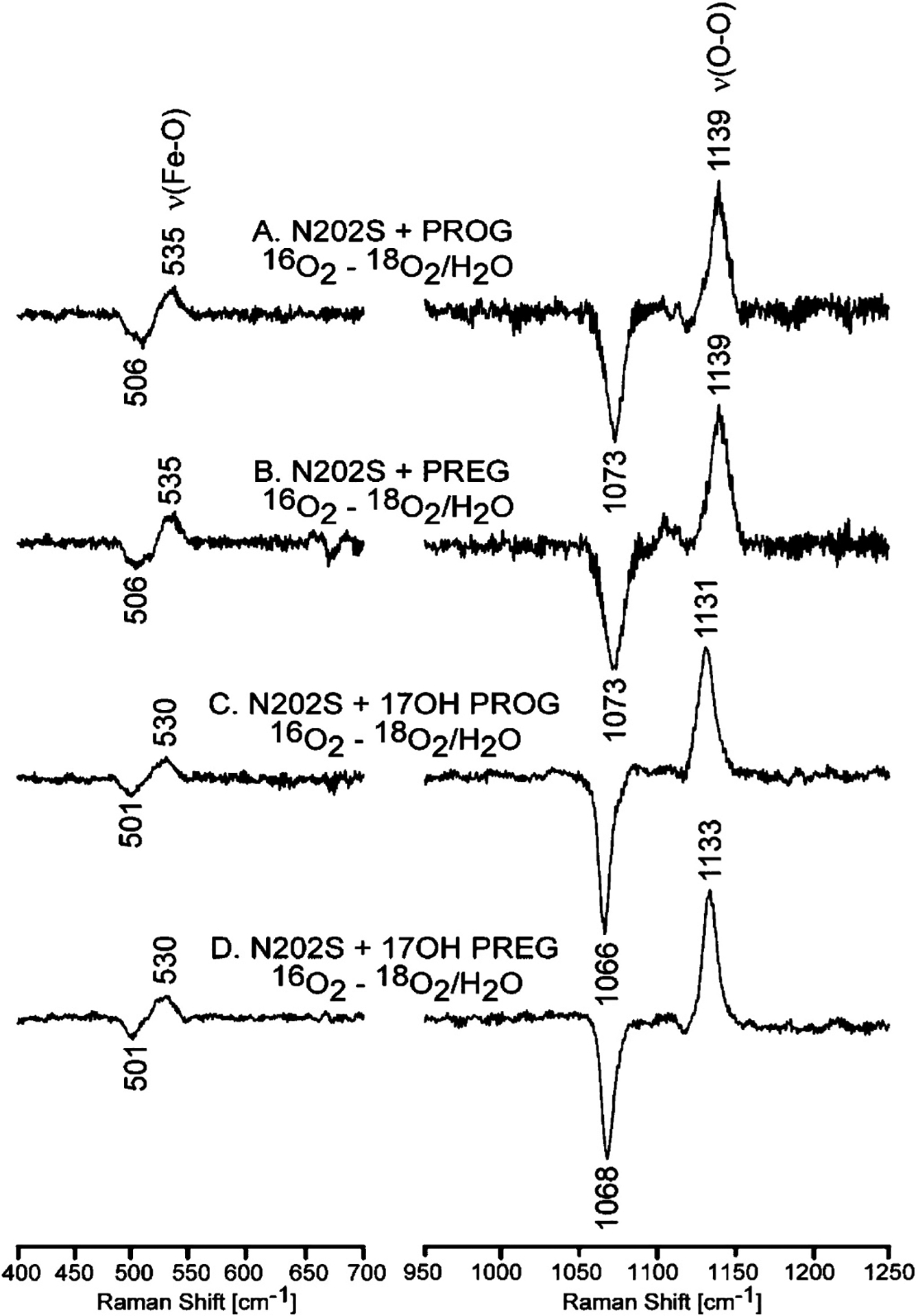
^16^O_2_ – ^18^O_2_ difference traces of the (A) PROG-, (B) PREG-, (C) 17-OH PROG-, and (D) 17-OH PREG-bound N202S CYP17A1 mutant in the low-frequency (left) and high-frequency (right) regions [124].

To gain insight into active site structural consequences of the N202S mutation, the rR spectra of oxy complexes with all four substrates were acquired (Fig. 14), for nonpolar substrates PROG and PREG, the wild type (WT) and N202S mutant display similar Fe-O-O core vibrations, with *ν*(Fe–O) near 535 cm^−1^ and ν(O–O) around 1140 cm^−1^. However, with hydroxylated substrates (17-OH PROG and 17-OH PREG), substantial changes in the ν(Fe–O) stretching modes were observed in N202S samples compared to WT; i.e., the 17-OH PROG sample exhibits ν(O–O) and ν(Fe–O) modes at 1131 and 530 cm^−1^, respectively, with expected ^16^O_2_ - ^18^O_2_ isotopic shifts (65 and 29 cm^−1^, respectively). A similar spectral pattern is observed for the 17-OH PREG-bound sample; the ν(O–O) stretching mode is observed at 1133 cm^−1^ and the ν(Fe–O) mode is observed at 530 cm^−1^, suggesting new hydrogen bonding interactions near the Fe-O-O fragment. More importantly, it is argued that the apparent substrate repositioning caused by the N202S mutation drastically alters the resultant interactions of the substrate-associated OH groups with the O^p^ and O^t^ atoms of the Fe-O^p^-O^t^ fragment, as is evident from the observed shifts of the *ν*(Fe-O) modes. In 17-OH PROG complexes, the mutant exhibits a 12 cm^−1^ lower *ν*(Fe–O) frequency (530 cm^−1^) compared to WT (542 cm^−1^), while in 17-OH PREG complexes, the *ν*(Fe–O) mode shifts 4 cm^−1^ higher (530 cm^−1^ vs. 526 cm^−1^ in WT). The downshift observed for 17-OH PROG in the mutant suggests a shift in hydrogen bonding of the C17-OH group from the terminal (O^t^) to the proximal (O^p^) oxygen, though less pronounced than that seen in WT with 17-OH PREG. These findings indicate that interactions between the substrates’ C-3 substituents and residue 202 influence substrate positioning in CYP17A1, providing compelling evidence that N202 plays a key role in orienting the C17-OH groups of 17-OH PROG and 17-OH PREG in the active site.

**Fig. 15.**
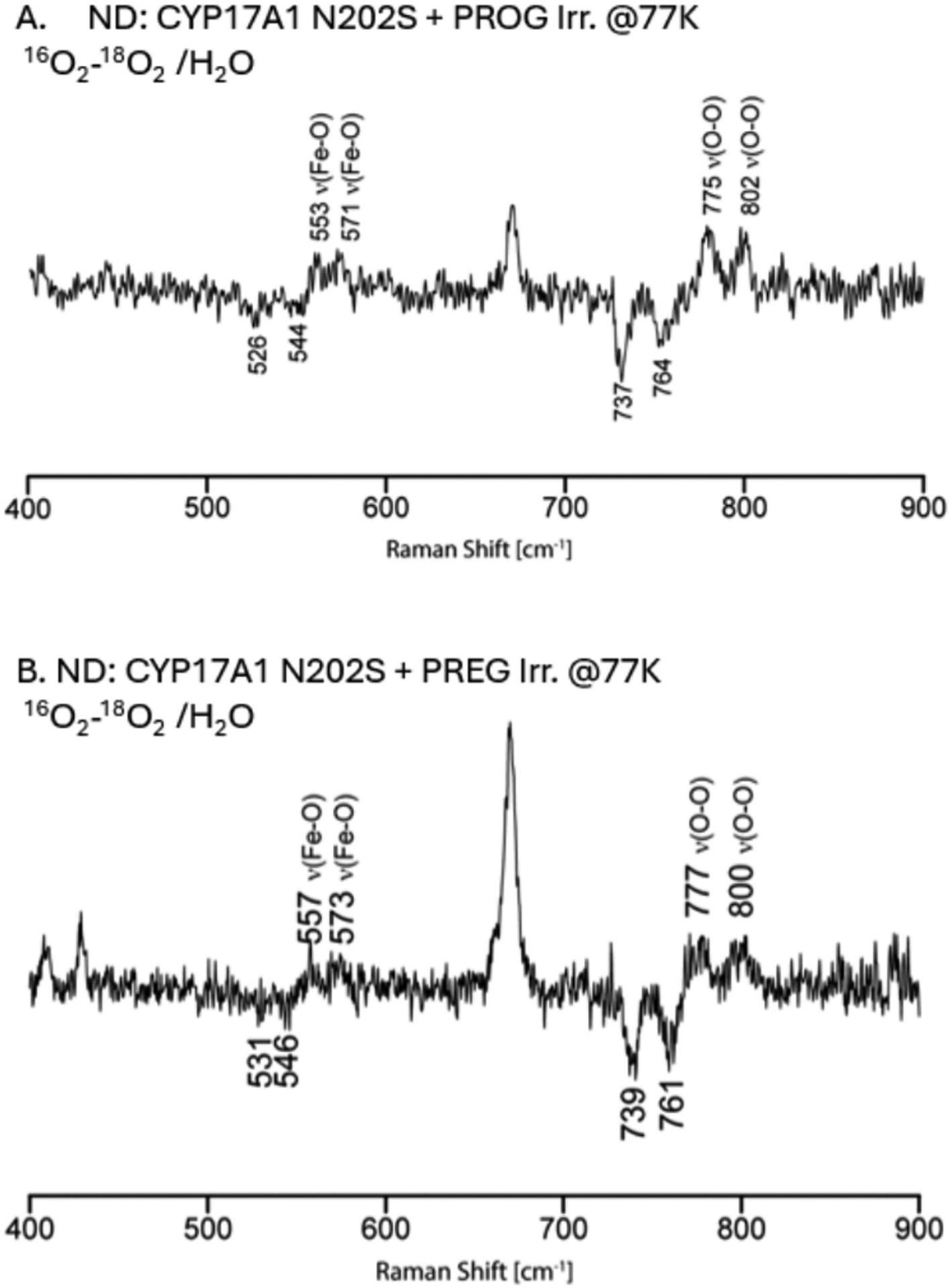
rR spectra data for irradiated dioxygen adducts of PROG (A) and PREG (B) bound CYP 17A1 (N202S mutant). Spectra were measured with 442.1 nm excitation at 77 K. Note that the peak near 674 cm^−1^ arises because of slightly different concentrations of the ^16^O_2_ and ^18^O_2_ intermediates, resulting in a residual heme internal mode in the positive side of the difference trace [24].

To investigate the structural factors underlying substrate specificity and lyase efficiency in CYP17A1, we conducted further rR analysis on irradiated and annealed samples of oxygenated N202S mutants [24]. The sample containing PREG (Fig. 15 A) exhibits two oxygen isotope-sensitive vibrational modes. One occurs at 802 cm^−1^ and shifts to 764 cm^−1^ upon substitution with ^18^O_2_, yielding an isotope shift of 38 cm^−1^. This value is consistent with a *ν*(O–O) stretching vibration and is most reasonably assigned to a peroxo-intermediate. The corresponding ν(Fe–O) mode is expected in the region where the feature at 553 cm^−1^ is observed, which shifts to 526 cm^−1^ in the ^18^O_2_ sample, as anticipated. Another ν(^16^O–^16^O) mode is observed at 775 cm^−1^, with a corresponding ν(Fe–O) mode at 572 cm^−1^; both display expected isotopic shifts upon ^16^O_2_–^18^O_2_ substitution (38 and 27 cm^−1^, respectively), confirming the identification of this species as the hydroperoxo derivative. The detection of both peroxo- and hydroperoxo-intermediates following cryoradiolysis of the dioxygen adduct of PROG-bound N202S CYP17A1 contrasts with results for the PROG-bound wild-type (WT) protein. In the WT, no evidence was found for the presence of the peroxo-intermediate, even at 77 K, with only the hydroperoxo species being observed. Thus, for the PROG-bound intermediate, protonation of the ferric peroxo-intermediate appears less efficient in the N202S mutant than in the WT protein.

Turning attention to the PREG-containing N202S sample (Fig. 15B), it shows two clear positive bands at 800 cm^−1^ and 777 cm^−1^ in the higher frequency region, both shifting as expected (39 and 38 cm^−1^) upon ^18^O_2_ substitution; these are most reasonably assigned to the *ν*(O–O) modes of the peroxo- and hydroperoxo- intermediates, respectively. The corresponding ν(Fe–O) modes are seen at 557 cm^−1^ (peroxo-) and 573 cm^−1^ (hydroperoxo-) with the expected isotopic shifts for the ^18^O_2_ containing samples. The overall behavior of these spectral patterns is almost identical to that observed for the WT enzyme in the presence of PREG [44], suggesting that the positioning of PREG in both WT and N202S mutant favors efficient proton transfer, which is required to facilitate the Compound I- mediated classical hydroxylation reaction producing 17-OH PREG.

**Fig. 16.**
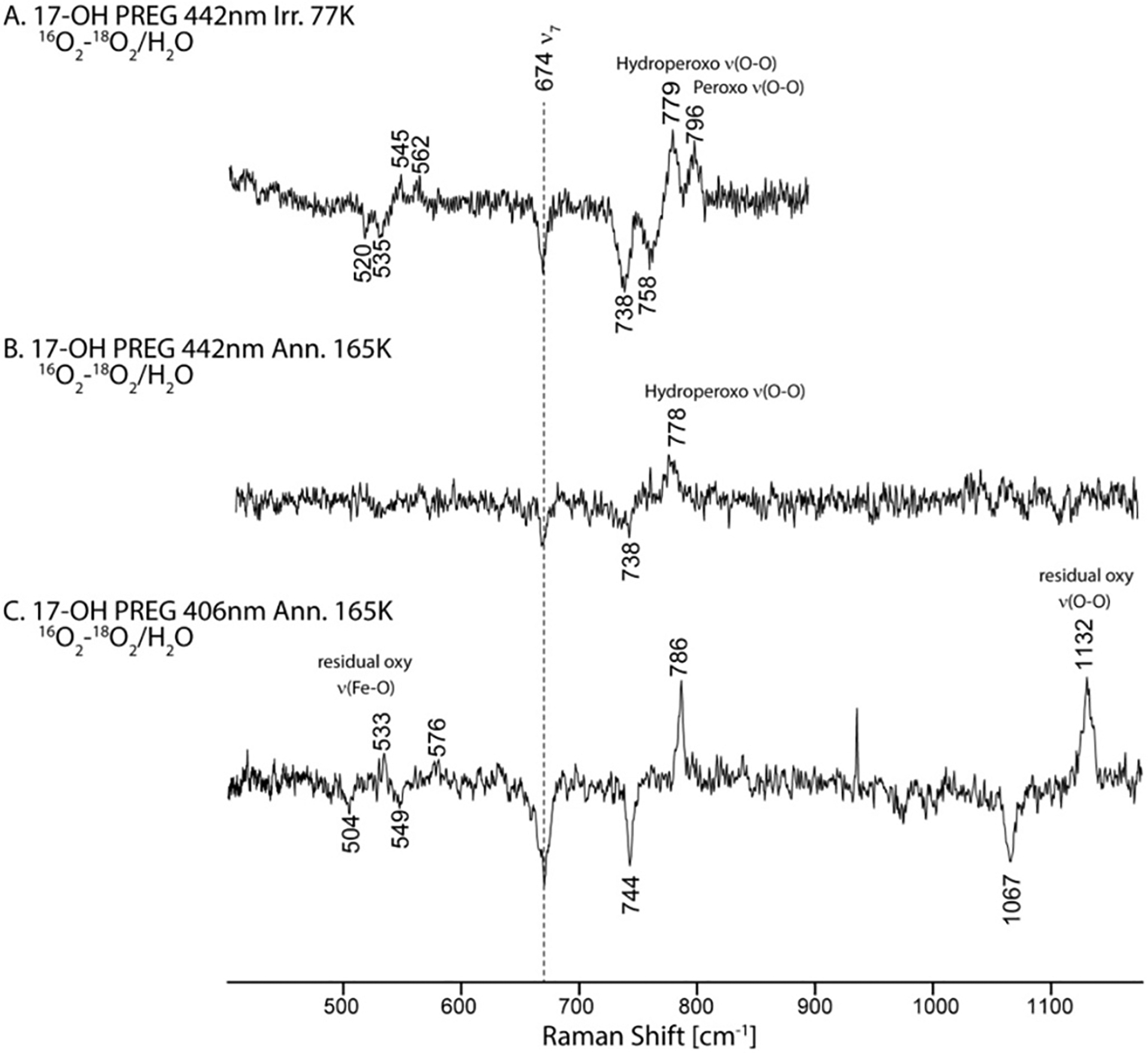
rR spectra data for irradiated dioxygen adducts of 17-OH PREG bound CYP17A1 (N202S mutant). Spectra were measured with 442.1 nm excitation line at 77 K and 165 K and with the 406.7 nm line of the sample annealed to 165 K. Note that the peak near 674 cm^−1^ arises because of slightly different concentrations of the ^16^O_2_ and ^18^O_2_ intermediates, resulting in a residual heme internal mode in the positive side of the difference trace [24].

The rR spectra obtained for the cryoreduced sample of the N202S CYP17A1 bound with the lyase substrate, 17-OH PREG, are shown in Fig. 16. The difference traces (Fig. 16 A) revealed the presence of both ferric peroxo- and hydroperoxo-intermediates. The peroxo-intermediate exhibited *ν*(^16^O–^16^O) and ν(Fe–^16^O) modes at 796 cm^−1^ and 545 cm^−1^, respectively, with isotope shifts consistent with prior assignments. Notably, unlike the wild-type enzyme (vide infra), the N202S mutant formed substantial amounts of the hydroperoxo species, characterized by modes at 779 cm^−1^ and 562 cm^−1^. The slightly lower *ν*(Fe–O) frequency for the hydroperoxo-intermediate in the 17-OH PREG-bound mutant, compared to other substrates, is attributed to hydrogen bonding between the 17-OH group and the proximal oxygen of the Fe–O–O fragment, an interaction that appears to persist upon protonation to form the hydroperoxo species. Note that the ν(Fe–O) mode for the hydroperoxo intermediate occurs approximately 10 cm^−1^ lower than those observed for other substrates (typically 571–576 cm^−1^). However, it should be remembered that the *ν*(Fe–O) mode for the peroxo intermediate in both the WT and mutant proteins appears 8–12 cm^−1^ lower in the PREG-bound sample. This shift is attributed to the 17-OH group forming a hydrogen bond with the proximal oxygen of the Fe-O^p^-O^t^ fragment. Therefore, we argue that this proximal H-bond persists upon protonation of the terminal oxygen to form the hydroperoxo-intermediate. As with the sample bound to the lyase substrate 17-OH PROG, rR spectra were acquired after annealing the cryoreduced oxy form of the N202S mutant, are shown in Fig. 16 A, revealing the trapping of both peroxo- and hydroperoxo-intermediates at 77 K, each exhibiting characteristic oxygen isotope-sensitive vibrational modes; i.e., the peroxo species exhibits a ν(O–O) mode at 789 cm^−1^ with the corresponding ν(Fe–O) mode occurring at 562 cm^−1^. The hydroperoxo species shows a ν(O–O) mode at 770 cm^−1^ with the corresponding ν(Fe–O) mode occurring at 576 cm^−1^.

**Fig. 17.**
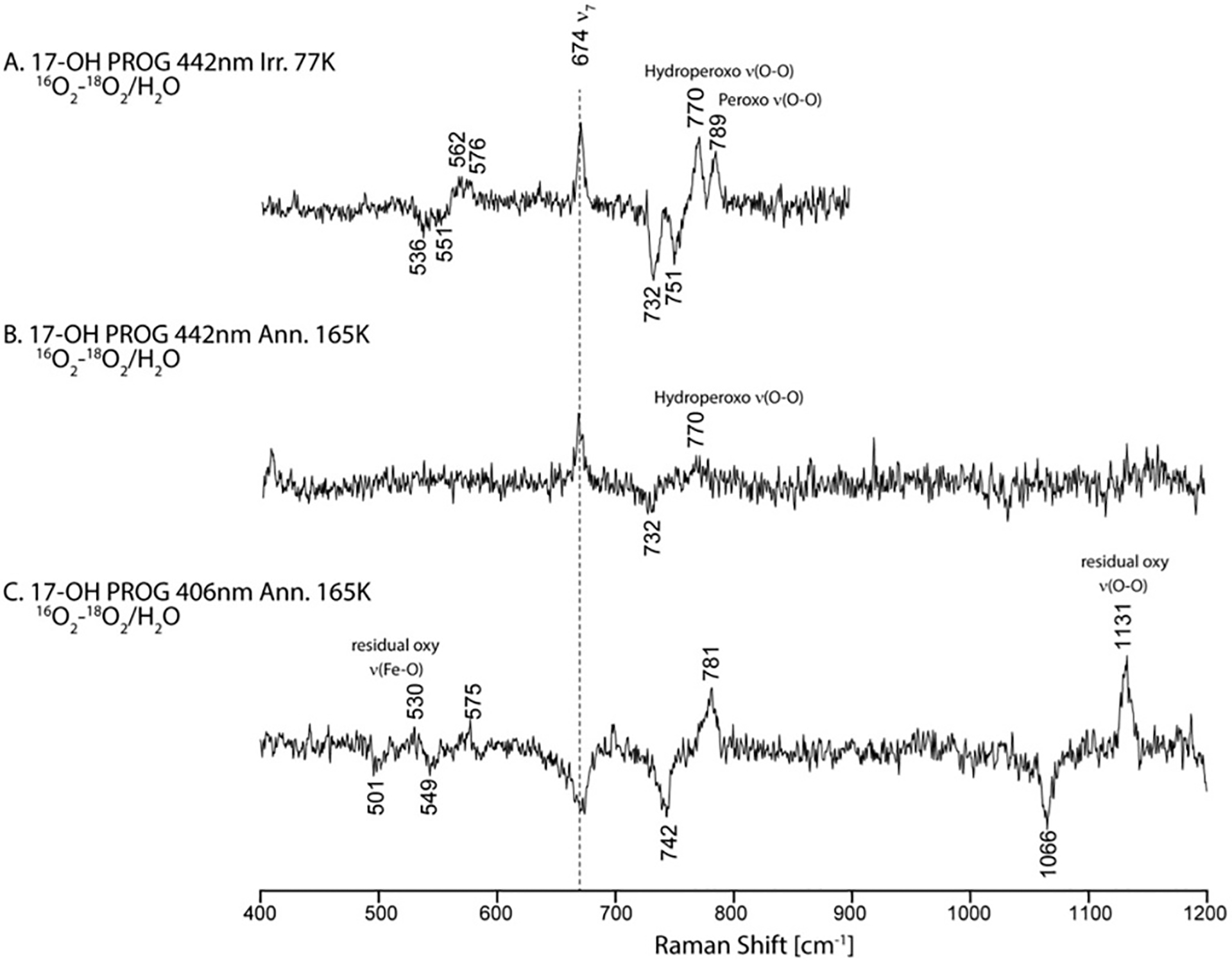
rR spectra data for irradiated dioxygen adducts of 17-OH PROG bound CYP17A1(N202S). Spectra were measured with 442.1 nm excitation line at 77 K and 165 K and with the 406.7 nm line of the sample annealed to 165 K. Note that the peak near 674 cm^−1^ arises because of slightly different concentrations of the ^16^O_2_ and ^18^O_2_ intermediates, resulting in a residual heme internal mode in the positive side of the difference trace [24].

Further annealing studies were conducted on the N202S mutant bound to the two lyase substrates, 17-OH PREG and 17-OH PROG. Upon annealing to 165 K, neither sample showed evidence of new ^16^O/^18^O-sensitive bands in the resonance Raman spectra (Fig. 16B and 17B), and the peroxo intermediate largely disappeared when using 442 nm excitation. The absence of new bands may result from conversion to a species with a different absorption maximum, as confirmed by separate UV–Vis experiments revealing a new intermediate absorbing at 405 nm. Indeed, resonance Raman measurements using 406 nm excitation detected a new intermediate in 17-OH PREG and 17-OH PROG bound N202S samples, exhibiting the set of ν(O–O)/*ν*(Fe–O) modes at 786/576 cm^−1^, and 781/575 cm^−1^, respectively (Figs. 16C and 17C). These features are associated with the proposed peroxo-hemiketal intermediate, as depicted in the center of the enzymatic cycle in Scheme 1. These spectral features are consistent with attack of the initially formed peroxo-intermediate at the C20 carbonyl of the lyase substrate, giving rise to the peroxo-hemiketal species depicted in Scheme 1, which eventually converts to the DHEA and AD product, respectively [44,45].

Importantly, kinetic solvent isotope effect (KSIE) experiments performed on the N202S mutant further support a peroxo-mediated catalytic mechanism. Inverse KSIE values were observed with both lyase substrates; i.e. 0.39 ± 0.03 with 17-OH PREG and 0.71 ± 0.01 with 17-OH PROG, consistent with a reaction pathway that bypasses the proton-dependent steps required for Compound I formation. These values are very close to the earlier results obtained for the WT CYP17A1 with the same substrates 0.39 [9] and 0.81 [45]. Notably, the inverse KSIE in 17-OH PROG sample is slightly more pronounced in the N202S mutant than in the WT, suggesting that the serine substitution at position 202 promotes a substrate orientation more favorable for peroxo-driven catalysis.

A key factor influencing lyase efficiency is the relative reactivity of the nucleophilic Fe-O-O peroxo- fragment, which is enhanced for systems possessing an H-bond donated to the proximal oxygen (O^p^) of the Fe-O^p^-O^t^ fragment as compared to the lowered nucleophilicity of an Fe-O^p^-O^t^ peroxo- fragment exposed to an H-bonding interaction at the terminal oxygen (O_t_) [15]. Inspection of Table 1 shows that the *ν*(Fe-O) and ν(O–O) modes in the N202S mutant are virtually identical to those in the WT enzyme, indicating that the hydrogen-bonding environment is largely preserved and that the nucleophilicity of the peroxo fragment is not significantly altered. However, other factors can also influence the lyase activity, including the relative efficiency of protonation of the reactive Fe-O-O fragment, producing the hydroperoxo intermediate, which could effectively divert the peroxo intermediate from the lyase cycle. As detected by rR spectroscopy, in the N202S samples, the most noticeable change compared to the WT protein is that the sample bound with 17-OH PREG provides evidence that the peroxo intermediate readily converts to the hydroperoxo species even at 77 K and continues to generate more of this species at 165 K, leaving no trace of the peroxo intermediate. Therefore, it is most reasonable to attribute the decrease in the lyase activity for the 17-OH PREG sample of the mutant to the increased efficiency of protonation, the consequence of which is to divert the reactive peroxo fragment from the lyase reactivity by generating the hydroperoxo intermediate. Collectively, these studies elucidate the role of the N202 residue in modulating substrate orientation and inducing active site structural changes that influence hydrogen bonding with the Fe–O–O fragment and, critically, the protonation state of the reactive ferric peroxo intermediate, thereby affecting lyase catalytic efficiency.

### 1.10. Functional and spectroscopic characterization of E305G/T306A mutant

As discussed earlier, in addition to the inherent reactivity of the ferric peroxo intermediate, the extent of protonation of the active site Fe-O-O fragment plays a critical role in regulating the CYP17A1 lyase reaction. Protonation leads to the formation of the hydroperoxo intermediate (Fe-O-O-H), effectively diverting the peroxo species away from the lyase pathway (Scheme 1) and thereby terminating its catalytic potential in C–C bond scission. It is important to note here that protonation of the active Fe-O-O fragment occurs via the proton shuttle network present in all cytochromes P450 [50] typically involving an acid/alcohol pair at the I-helix. In the case of CYP17A1, this acid/alcohol pair is associated with the E305 and T306 residues [42,62,63]. The rR spectroscopic studies of cryoradiolytically generated intermediates are well suited to document this protonation, providing direct interrogation of the H-bonding interaction and extent of protonation of the ferric-peroxo species via the measured relative intensities of the peroxo- and hydroperoxo- rR signals.

Recognizing the power of this methodology, we further explored the structural basis for functional effects observed upon introducing key active site mutations of the acid/alcohol proton shuttle of CYP17A1, which is considered to be necessary for efficient proton delivery in the enzyme catalytic cycle [41,42]. Earlier we have shown that the T306A mutation of CYP17A1, the analogous position to CYP101’s T252 residue, had a dramatic effect on the hydroxylation activity but a much smaller effect on the lyase reaction, suggesting the Thr306 involvement in a proton delivery pathway Compound I formation [10]. To further document the effect of the T306A mutation on both hydroxylase and lyase reactions, rR spectroscopy was employed to evaluate the associated structural changes within the active site.

**Fig. 18.**
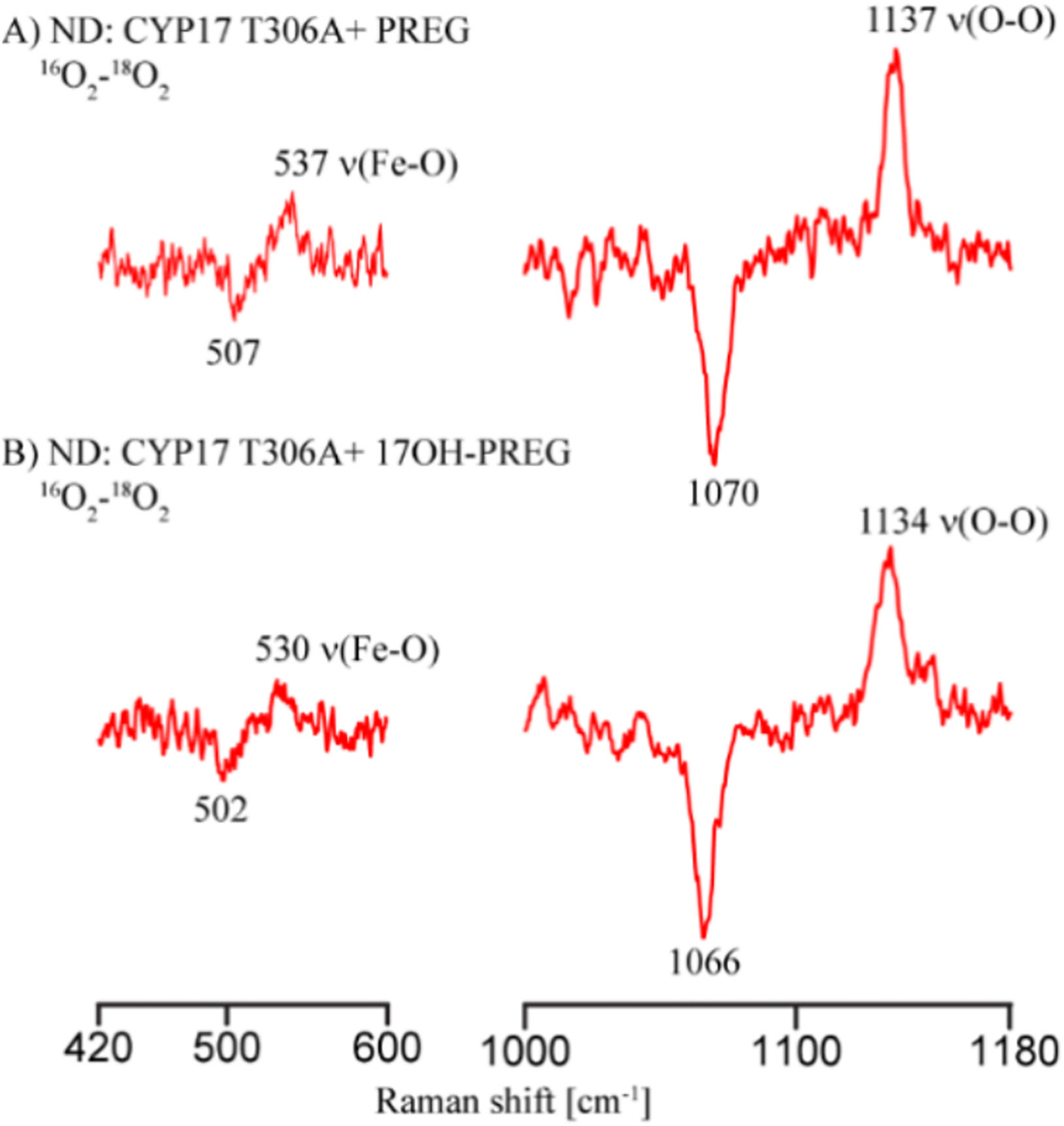
The rR spectra of ^16^O_2_–^18^O_2_ difference plots of CYP17A1 T306A samples with PREG (A) and 17-OH PREG (B). Spectra were measured using the 413.1 nm excitation [41].

The rR spectra of dioxygen adducts of the CYP17A1 T306A mutant revealed distinct isotope-sensitive vibrational features for both PREG-and 17-OH PREG-bound forms (Fig. 18). The *ν*(O–O) and ν(Fe–O) modes observed in each complex were consistent with predicted isotopic shifts. Importantly, the ν(Fe–O) mode in the 17-OH PREG-bound sample (530 cm^−1^) was downshifted by approximately 7 cm^−1^ relative to the PREG-bound form (537 cm^−1^), indicating a hydrogen-bonding interaction with the proximal oxygen (O^p^) of the Fe-O^p^–O^t^ fragment. Such behavior is similar with that observed for the wild-type CYP17A1 protein [43,44], where the ν(Fe-O) mode of hydroxylated substrates is 9 cm^−1^ downshifted from that for the non-H-bonding PREG sample. These results suggest that, despite potential alterations to the active site architecture resulting from the T306A mutation, a key hydrogen-bonding interaction with the proximal oxygen is retained. This interaction plays a significant role in modulating the electronic structure and reactivity of the Fe-O-O fragment, with important implications for the catalytic function of CYP17A1.

Irradiated samples of CYP17A1 T306A bound with PREG generate a peroxo species, as indicated by rR spectra that exhibit a set of oxygen isotope-sensitive modes. A ν(^16^O–^16^O) mode is observed at 811 cm^−1^, and the corresponding ν(Fe–^16^O) mode appears at 552 cm^−1^, as summarized in Table 1. Annealing of the sample containing the PREG to 165 K reveals a lack of hydroperoxo intermediate formation upon annealing to 165 K, with only signals from residual unreduced dioxygen adducts observed. This contrasts sharply with wild-type CYP17A1, which undergoes efficient conversion from peroxo to hydroperoxo species under similar conditions. The results are consistent with previous findings for Thr to Ala mutations in P450 enzymes, such as T252A in CYP101, which disrupt proton delivery and destabilize the hydroperoxo intermediate, ultimately favoring non-productive decay pathways and hydrogen peroxide release [50,85,86,125].

**Fig. 19.**
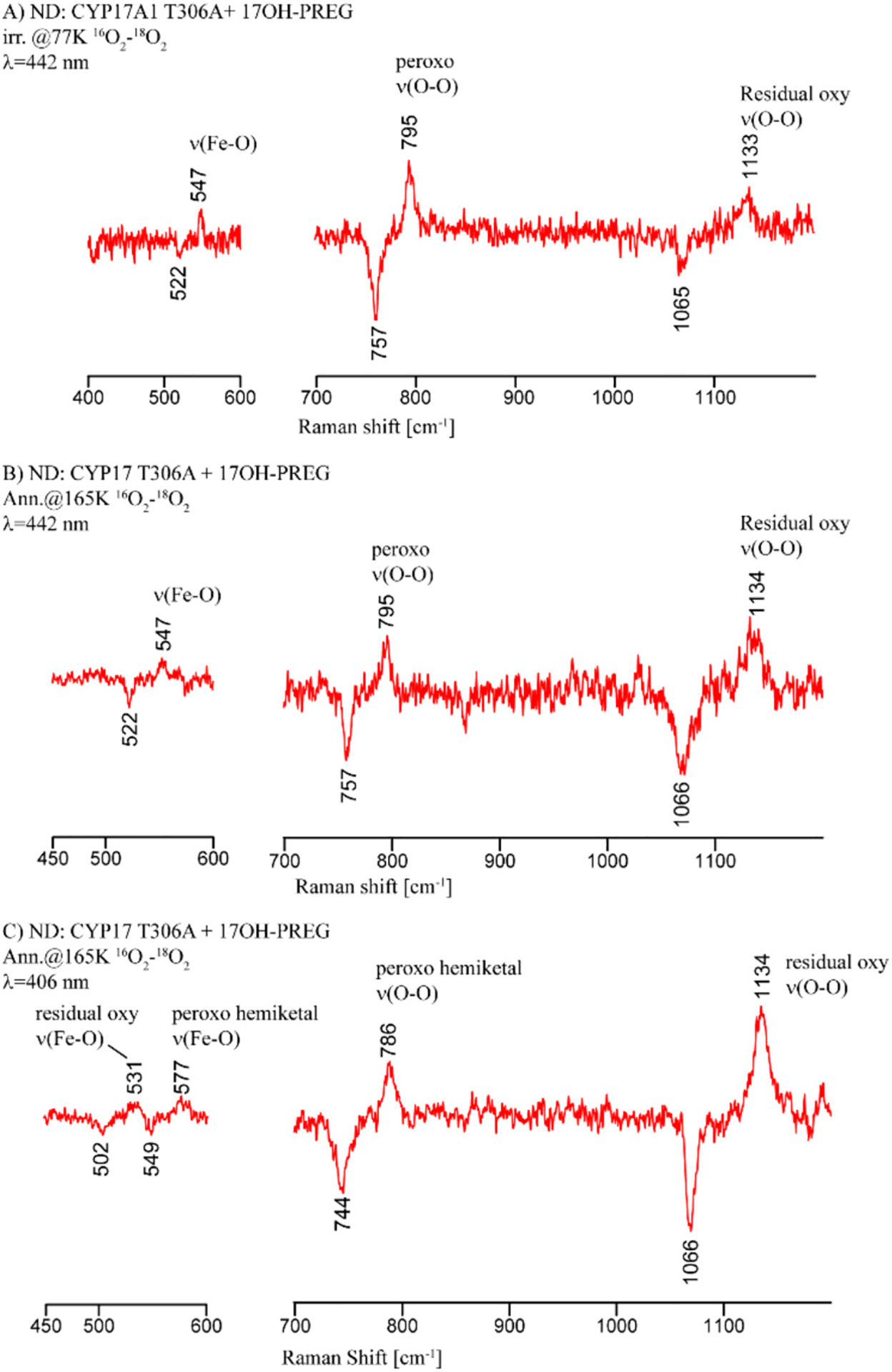
rR difference spectra data for irradiated and annealed dioxygen adducts of 17-OH PREG bound CYP 17A1(T306A mutant). (A) The rR difference spectrum of irradiated oxy samples. (B) The corresponding samples after annealing to 165 K and spectra were measured with 442 nm excitation line. (C) The corresponding samples after annealing to 165 K, spectra were measured with 406.7 nm excitation [41].

In contrast, with the susceptible lyase substrate, 17-OH PREG, the rR spectra (Fig. 19 A) not only reveals an initially trapped peroxo-iron intermediate experiencing an H-bond interaction of the 17-OH group with the proximal oxygen of the Fe-O^p^-O^t^ fragment, facilitating peroxo-attack on the C20 carbon, but also shows the presence of the subsequent hemiketal intermediate of the lyase reaction upon further annealing to 190 K (Fig. 19C), a species which spontaneously converts to the DHEA lyase product [41]. These results provide additional evidence for the peroxo-mediated C_17_-C_20_ scission mechanism of 17-OH PREG in CYP17A1.

**Fig. 20.**
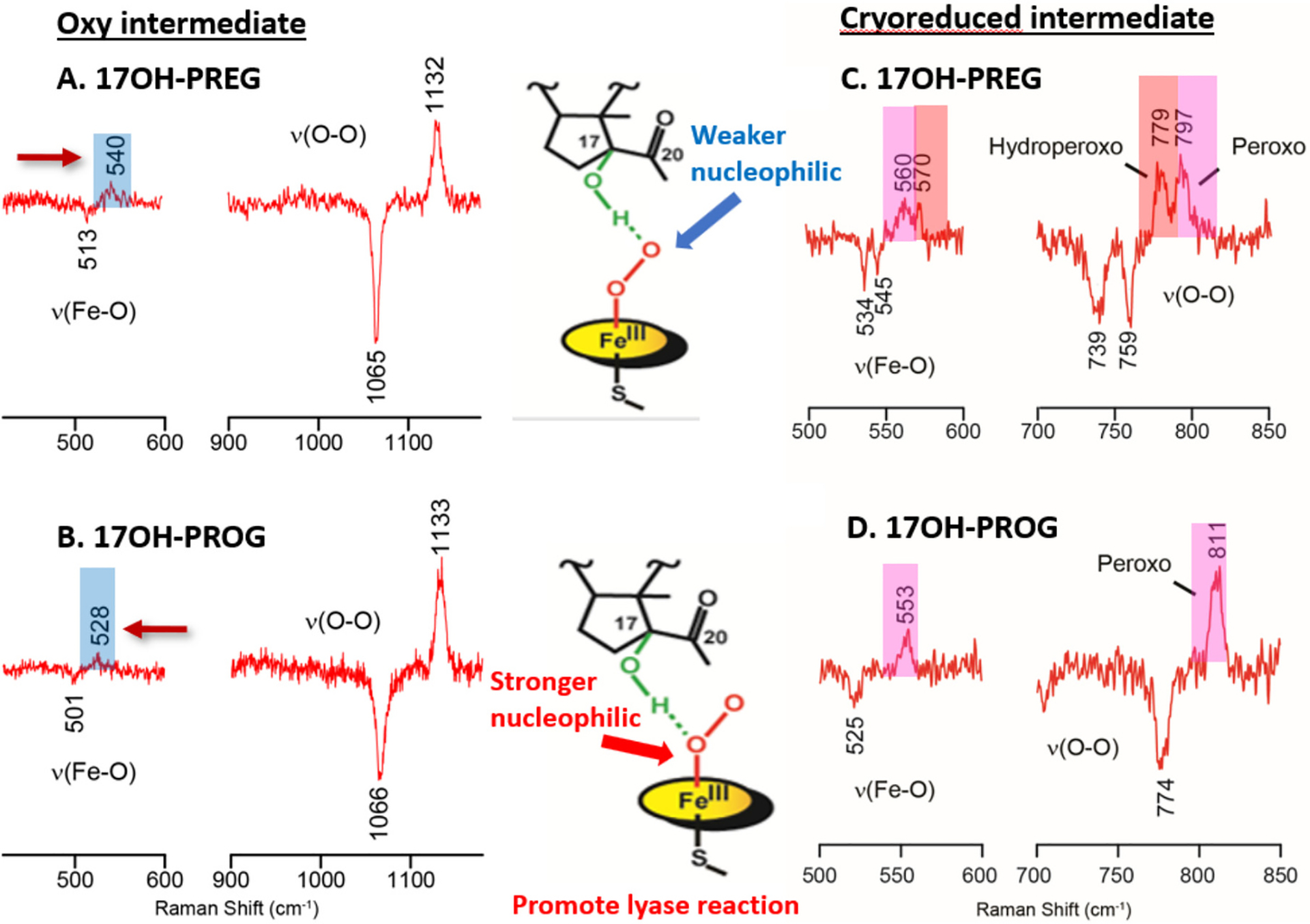
Resonance Raman spectral data for irradiated dioxygen adducts of CYP17A1 E305G mutant. The rR spectra of oxy intermediates were measured with 413 nm excitation line, the cytoreduced intermediates are measured with 442 nm excitation line at 77 K. The rR ^16^O_2_–^18^O_2_ difference traces of oxy CYP17A1 E305G samples with 17-OH PREG (A) and 17-OH PROG (B) and corresponding irradiated samples (C) and (D) [42].

Analogous spectroscopic and functional studies were performed on the clinically relevant Glu305Gly mutant (E305G), which decreases the activity with 17-OH PREG (Δ5) as substrate but increases the activity of 17-OH PROG (Δ4) carbon-carbon bond scission, compared with the wild-type enzyme [42]. Strikingly, the E305G mutation reverses both the rates of product formation and the Δ4/Δ5 pattern in the measured solvent isotope effects. For the WT CYP17A1 faster C–C bond scission was observed with 17-OH PREG as a substrate with the rate 0.76 min^−1^ vs. 0.34 min^−1^ for 17-OH PROG, with corresponding KSIE values 0.39 and 0.80. The same rates measured for the E305G mutant are 0.21 min^−1^ (17-OH PREG) and 0.48 min^−1^ (17-OH PROG) with KSIE values 0.80 and 0.54. This dramatic reversal of efficiencies for C–C bond cleavage by replacement of a proton shuttle residue is quite intriguing. In order to gain insight into active site structural consequences of the E305G mutation, the rR spectra of the oxy complexes with the 17-OH PROG and 17-OH PREG substrates were acquired and showed substantial changes in the *ν*(Fe–O) stretching modes relative to the WT enzyme (Fig. 20A and B) [42]. With wild type, the substrate 17-OH PREG provides an H-bond to the proximal oxygen, exhibiting a ν(Fe–^16^O) at 526 cm^−1^, whereas for the E305G variant, this same substrate is oriented to generate an H-bonding interaction primarily with the terminal oxygen, exhibiting a ν(Fe–^16^O) at 540 cm^−1^. Conversely, for the 17-OH PROG complex in the E305G mutant, an H-bonding interaction with the proximal oxygen is documented, with a ν(Fe–^16^O) appearing at 528 cm^−1^, whereas the corresponding complex with the wild-type enzyme generates an H-bonding interaction with the terminal oxygen; i.e., ν(Fe–^16^O) at 542 cm^−1^ (Table 1). Thus, the rR data for E305G and wild-type enzyme show that the H-bond interactions with the Fe-O-O fragment are reversed, and such altered H-bonding interaction seen in the dioxygen adduct does persist in the peroxo species (Fig. 20C and D). These rR results clearly show that the E305G mutation changes the orientation of the lyase substrate in the active site, which alters a critical hydrogen bonding of the 17-alcohol to the iron-bound peroxide. The observed switch in substrate specificity of the enzyme is consistent with this result if the hydrogen bonding to the proximal peroxo oxygen is essential for a proposed nucleophilic peroxoanion-mediated mechanism for CYP17A1 in carbon-carbon bond scission.

Our functional studies demonstrated that hydroxylation and lyase reactions proceed via different catalytic pathways. Mutations at the acid-alcohol pair E305G and T306A significantly perturb hydroxylation activity, with the product formation rate decreasing 10–15 fold, similarly to other cytochromes P450 [10,42]. The lyase reactions were not significantly affected by these mutations. In addition, the pH dependence of the lyase reactions was much stronger in both original CYP17A1 and the one with E305G mutation than for hydroxylation reactions, also indicating a different catalytic mechanism. Notably, the spectroscopic data provide evidence for both the peroxo intermediate and the subsequent lyase-specific hemiketal intermediate, formed independently of proton delivery in the active site. These findings confirm the catalytic mechanism involving a nucleophilic peroxo-anion rather than the traditional Compound 1 as the active oxidant.

### 1.11. The role of protein-protein interactions in the control of lyase chemistry of CYP17A1

In addition to characterization of catalytic intermediates as described above, a critical aspect of P450 function is interaction with redox partners for efficient electron transfer. The detailed kinetic studies of the CYP17A1/Cytb_5_ complex in Nanodiscs revealed that the presence of Cytb_5_ accelerates the lyase activity by ~5-fold for 17-OH PREG and by ~7-fold for 17-OH PROG, displaying a heretofore unexplained more pronounced acceleration when 17-OH PROG is a substrate, with the origin of the stimulating effect of Cytb_5_ on CYP17A1 lyase reaction is still in debate. One possibility is that Cytb_5_ acts as a complementary electron donor, working in concert with CPR to more efficiently complete the lyase cycle [38] with the first electron transfer requiring CPR and the second electron being donated by either CPR or Cytb_5_ [1,74,126].Clearly, the presence of a reduced Cytb_5_ donor positioned near the newly formed oxygenated intermediate could offer a significant advantage.

**Fig. 21.**
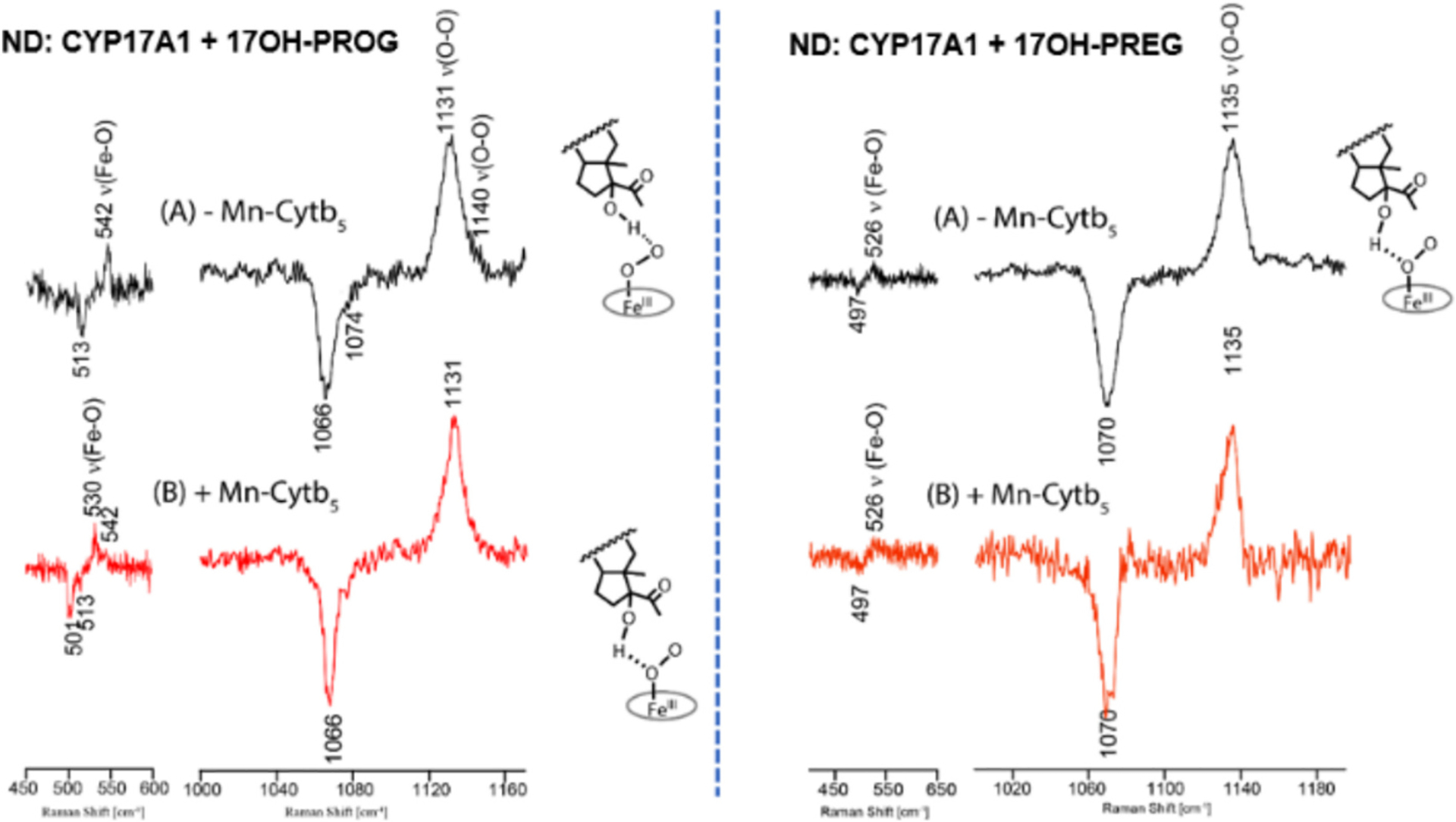
^16^O_2_–^18^O_2_ rR difference spectra of 17-OH PROG bound CYP17A1 oxy complex without (A) and with Mn Cytb_5_ (B) [75].

Given the profound functional effects of Cytb_5_ on CYP17A1 catalytic activity, we have performed a rR study of these assemblies; i.e., CYP17A1/Cytb_5_, where the Mn-Porphyrin-substituted Cytb_5_ was employed whose allosteric effects are similar to native Cytb_5_ but does not have its rR-active electronic absorption band overlapped with that of CYP17A1 [75]. The rR studies of these CYP17A1/Mn-Cytb_5_ complexes in Nanodiscs provided the first spectroscopic evidence of active site structure changes due to the allosteric interactions with Cytb_5_. Specifically, we compared the effect of Mn-Cytb_5_ on the oxy complex of CYP17A1 with both proteins co-incorporated in Nanodiscs. For CYP17A1 with 17-OH PROG, the rR spectrum of the sample including Mn-Cytb_5_ reveals an active site rearrangement yielding a dominant fraction of the oxy complex possessing a *ν*(Fe–^16^O) mode at 530 cm^−1^ compared to 542 cm^−1^, a spectral shift consistent with transfer of the H-bonding interaction from the terminal to the proximal oxygen of the Fe-O-O fragment (Fig. 21, left panel). This is significant, because this configuration favors the lyase catalysis, i.e., the conformer well oriented to form the peroxo–hemiketal intermediate. In contrast, for 17-OH PREG, no such shift is observed, the favorable H-bonding orientation being present even without Mn-Cytb_5_ (Fig. 21, right panel). The additional enhancement of lyase activity seen for 17-OH PROG compared to 17-OH PREG is shown by these Raman experiments to be attributed to an allosteric effect of Cytb_5_ which shifts the H-bond interaction of the 17OH group from the terminal to the proximal oxygen of the Fe-O-O fragment. This result highlights the impressive utility of rR spectroscopy to detect quite subtle, but functionally important, changes in active site architecture induced by crucial intermolecular interactions.

In summary, in this review we describe our efforts to understand the detailed catalytic mechanisms of the human steroid metabolizing P450 CYP17A1 by combining biochemical functional studies with detailed spectroscopic characterization of catalytic intermediates. In all of our experiments we used CYP17A1 self-assembled into a lipid bilayer Nanodisc in order to provide a native-like membrane environment for this heme enzyme and its redox partners cytochrome P450 reductase and cytochrome *b*5. This stable monomeric structurally homogeneous environment allow formation and stabilization of 100% pure oxy-complexes of CYP17A1 saturated with selected substrates. Critical was the use of resonance Raman spectroscopy. The isotopic H/D and ^16^O/^18^O substitutions allow identifying vibrational bands of the Fe-OO moiety in resonance with the heme Soret band. Resonance Raman spectroscopy provides structural assignment for the position of all four substrates with respect to the Fe-OO moiety and allow identifying the specific catalytically important hydrogen bonds of C17-OH group to the proximal and/or distal oxygen atoms. Selective mutations are used to confirm assignment and to reveal the mechanistic origin of functional alterations. For instance, the N202S mutation changes the position of the substrates PROG and PREG leading to the partial reversal of this hydrogen bonding pattern with concomitant functional implications.

Cryoradiolytic reduction of oxygenated CYP17A1 allows one to generate and trap a high yield of the peroxo- and hydroperoxo- ferric intermediates. Being stable at liquid nitrogen temperature, they can be probed by optical and resonance Raman spectroscopy. Gradual annealing, to 165 K and higher temperatures, allows the reaction to proceed, showing protonation of these intermediates and subsequent generation of either a catalytically active Compound 1 or transient hemiketal intermediate, with subsequent product formation. These processes were also documented spectroscopically, and comparing the wild-type enzyme to the functionally important mutations E305G and T306A allows further support for mechanistic conclusions. A unique spectral signature of the hemiketal intermediate, the trapped intermediate following peroxo anion attack, was therefore revealed for the lyase activitiy of native and mutant CYP17A1 with both 17-OH PROG and 17-OH PREG as substrates.

Consistent with the two alternative catalytic pathways for hydroxylation and C–C bond scission are the solvent isotope effects measured for these two reactions. The normal KSIE for hydroxylation was obtained with WT CYP17A1 and all studied mutants, while inverse KSIE was always measured in all cases when lyase substrates 17-OH PROG and 17-OH PREG were used. The inverse KSIE confirmed that C–C bond scission catalysis does not require protonation of the peroxo-iron intermediate, meaning that this reactions goes mostly via alternative pathway with little or no product forming by Compound 1.

The stopped-flow experiments monitoring the decay of oxy-complex in CYP17A1 in the presence of reductase and/or cytochrome *b*5 demonstrated the significant role of the latter in the second electron transfer to oxygenated CYP17A1. The complex of CYP17A1 with cytochrome *b*5 in Nanodiscs was also characterized by rR spectroscopy, and the shift of the Fe–O band in the complex with 17-OH PROG was detected, indicating the allosteric effect of Cytb_5_. Therefore, the effect of cytochrome *b*5 on the CYP17A1 catalytic activity includes both the acceleration of reduction of oxy-complex and the allosteric conformational perrurbation of substrate positioning with respect to the Fe-OO moiety.

In summary, this multi-investigator collaboration over many years brought together a broad spectrum of techiniques that, together, allowed a comprehensive understanding for the multiple catalytic mechanisms of the ubiquitous cytochrome P450 superfamily.

## Figures and Tables

**Table 1:** Summary of the *ν*(Fe–O) and ν(O–O) vibrational modes in different intermediates for CYP17A1 wild-type, mutants (N202S, E305G, and T306A), and the complex of CYP17A1 with cytochrome *b*_5_.

	Oxy Fe^3+^-OO^−^		Peroxo Fe^3+^-OO^2−^		Hydroperoxo Fe^3+^-OOH^−^		Hemiketal intermediate (Scheme 1, center)	
	*ν*(Fe—O)	*ν*(O—O)	*ν*(Fe—O)	*ν*(O—O)	*ν*(Fe—O)	*ν*(O—O)	*ν*(Fe—O)	*ν*(O—O)
**WT CYP17A1**								
PROG	536	1140	no	no	575	772	no	no
PREG	535	1140	554	802	572	775	no	no
17-OH PROG	542	1131	562	790	576	771	573	785
17-OH PREG	526	1135	546	796	no	no	579	791
**N202S CYP17A1**								
PROG	535	1139	553	802	571	775	no	no
PREG	535	1139	557	800	573	777	no	no
17-OH PROG	530	1131	562	789	576	770	575	781
17-OH PREG	530	1133	545	796	562	779	576	786
**E305G CYP17A1**								
17-OH PROG	528	1133	553	811	570	790	no	no
17-OH PREG	540	1142	560	797	no	no	571	786
**T306A CYP17A1**								
PREG	537	1137	552	811	no	no	no	no
17-OH PREG	530	1140	547	795	no	no	577	786
**CYP17A1 + Cyt b5**								
17-OH PROG	542	1131						
17-OH PREG	526	1135						

Note: NO indicates not observed.
